# FOXP3 full length splice variant is associated with kidney allograft tolerance

**DOI:** 10.3389/fimmu.2024.1389105

**Published:** 2024-04-10

**Authors:** Qais W. Saleh, Afsaneh Mohammadnejad, Martin Tepel

**Affiliations:** ^1^ Department of Nephrology, Odense University Hospital, Odense, Denmark; ^2^ Cardiovascular and Renal Research, Department of Molecular Medicine, University of Southern Denmark, Odense, Denmark; ^3^ Epidemiology, Biostatistics and Biodemography, Department of Public Health, University of Southern Denmark, Odense, Denmark

**Keywords:** kidney transplantation, forkhead box P3, splice isoforms, immunosuppression, biomarker, glomerular filtration rate

## Abstract

**Background:**

Progressive decline of allograft function leads to premature graft loss. Forkhead box P3 (FOXP3), a characteristic gene of T-regulatory cells, is known to be essential for auto-antigen tolerance. We assessed the hypothesis that low FOXP3 mRNA splice variant levels in peripheral blood cells early after transplantation are associated with progressive allograft injury.

**Methods:**

Blood samples were prospectively collected from 333 incident kidney transplant recipients on the first and 29th postoperative day. We used quantitative polymerase chain reaction to determine transcripts of 3 isotypes of FOXP3 splice variants, including pre-mature FOXP3 and full length FOXP3 (FOXP3fl). We investigated the association between FOXP3 splice variant levels and the declines in estimated glomerular filtration rate (eGFR) of more than 5ml/min/1.73m^2^ within the first-year post-transplant using logistic regression.

**Results:**

We observed lower FOXP3fl levels in recipients with declining eGFR (N = 132) than in recipients with stable eGFR (N = 201), (logarithmic value -4.13 [IQR -4.50 to -3.84] vs -4.00 [4.32 to -3.74], p=0.02). In *ad hoc* analysis pre-transplant FOXP3fl levels were similar in both groups. The association between FOXP3fl and declining eGFR was confirmed by multivariable analysis adjusted for potential confounding factors (Odds Ratio 0.51, 95% confidence interval 0.28 to 0.91: p=0.02). When stratifying FOXP3fl levels into quartiles, recipients with lower day1 FOXP3fl had the highest rate of declining eGFR (p=0.04).

**Conclusion:**

Low FOXP3fl splice variant levels at the first postoperative day in kidney transplant recipients were associated with severe decline of eGFR, a well-known surrogate for hard endpoints.

## 1 Background

Progressive decline of kidney allograft function is an important problem which affects patients’ morbidity, mortality, quality of life, and may lead to early return to dialysis (1). However, there is a lack of a validated and reproducible method to identify recipients with higher capacity to tolerate transplanted kidney allografts.

Levels of the forkhead box P3 (FOXP3) mRNA may reflect a recipient’s capacity to tolerate transplanted tissues. FOXP3 is a transcription factor which is considered the master gene regulator for T-regulatory cells as the expression of FOXP3 induces the phenotype and function of these cells (2, 3). T-regulatory cells exhibit immunosuppressive capabilities and infer immunologic tolerance to auto- and alloantigens (4, 5). In kidney transplant recipients, studies have shown that recipients with higher levels of T-regulatory cells are less likely to experience allograft rejection, and that recipients with higher levels of FOXP3 show stable allograft function despite negligible immunosuppressive therapy (6). Moreover, FOXP3 levels are altered by immunosuppressive therapy (6) and could mirror a recipient’s degree of immunosuppression. A few studies have measured FOXP3 mRNA levels in kidney transplant recipients, but these did not include the different FOXP3 splice variants (7, 8).

Unlike in mice, FOXP3 is spliced into three mRNA isotypes by mRNA splicing in humans. The major variants, which account for up to 99 percent, are full length FOXP3 (FOXP3fl) in which all exons are expressed and FOXP3 lacking exon 2 (FOXP3d2) (9). FOXP3fl and FOXP3d2 have been described to account for up to 30% and 70% of all transcribed FOXP3 mRNA in human T regulatory cells, respectively (9). These major variants are co-expressed in normally functioning T regulatory cells (10, 11). Exon two partly encodes a nuclear export factor and a repressor domain (12, 13). The presence of a nuclear export factor enables FOXP3fl to translocate to the cytoplasm (12), which suggests that FOXP3fl exerts isoform specific functions in the cytoplasm (9, 12). In addition, evidence suggests that FOXP3fl and FOXP3d2 exert isoform specific gene regulatory activities. T regulatory cells that lack FOXP3fl expression exhibit altered expression of proteins that influence T regulatory cell stability, such as ID3, Bcl6 and eIF4E (14). Furthermore, lack of FOXP3fl also results in decreased expression of CD25, which is essential for T regulatory cell stability.

We tested the hypothesis that lower FOXP3 mRNA splice variant levels in kidney transplant recipients are associated with declining kidney allograft function.

## 2 Methods

### 2.1 Setting and ethical approval

This observational study is an ancillary study of the molecular monitoring after kidney transplantation project (MoMoTx) (15, 16). Shortly, MoMoTx is an ongoing single-center prospective project which includes incident kidney transplant recipients aged 18 or older who are transplanted at Odense University Hospital, Denmark. Recruitment of participants has been ongoing since January 2011. Patients who receive re-transplantations are included and registered as new participants.

The study protocol is in accordance with the ethical standards of the Declarations of Helsinki and Istanbul, and has been approved by the local ethics committee (Den Videnskabsetiske Komite for Region Syddanmark, Project-ID: 20100098). All participants gave informed consent before entry into the study. Blood samples are collected from each participant before and after transplantation.

### 2.2 Blood samples and clinical data

**Figure 1:**
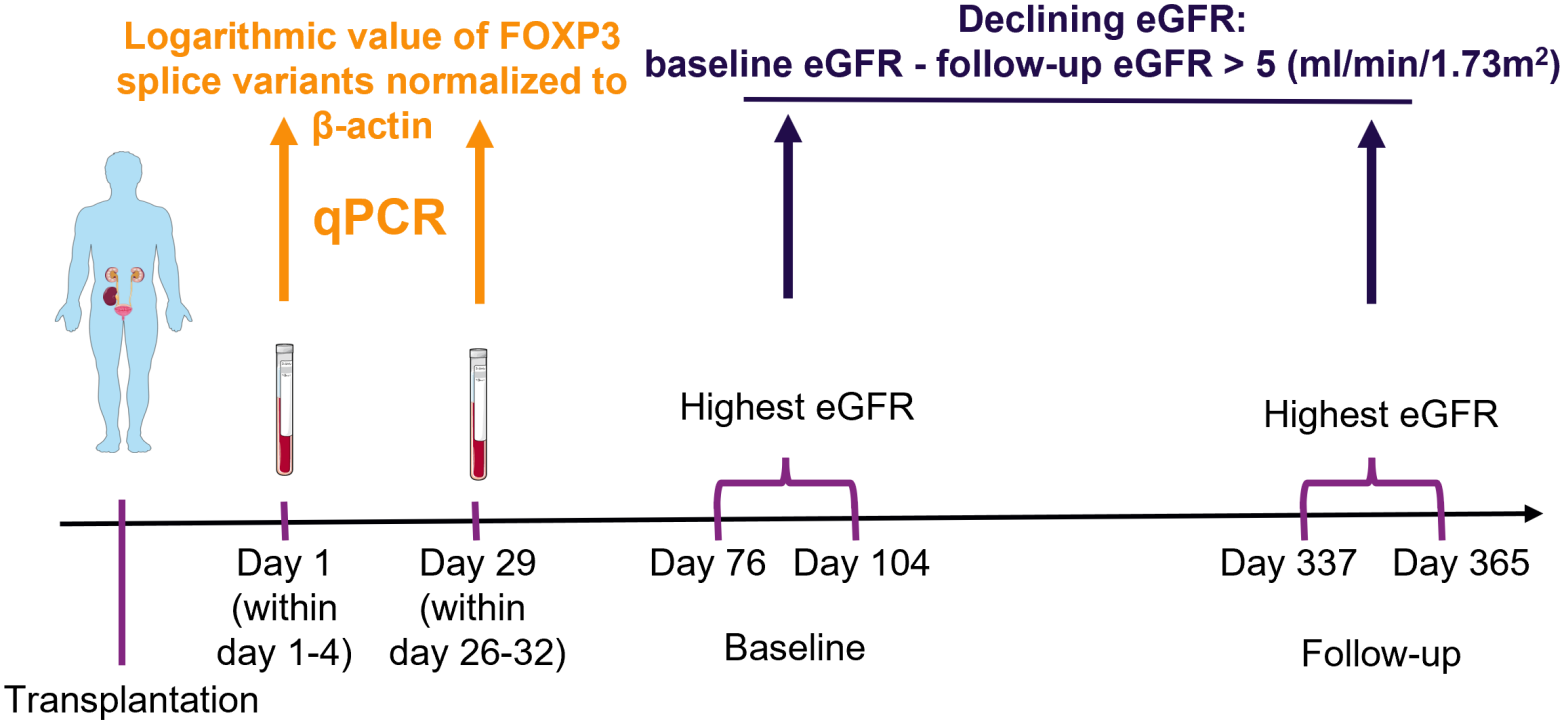
Schematic presentation of methodology. Blood samples were collected from kidney transplant recipients, ribonucleic acid was extracted and used in quantitative polymerase chain reaction (qPCR) to measure the exposure variables of interest: FOXP3 splice variants. The highest creatinine value measured at baseline and follow-up were used to determine the estimated glomerular filtration rate (eGFR) with the CKD-EPI formula. The outcome was defined as a decrease from baseline to follow-up larger than 5 ml/min/1.73m^2^. Finally, potential association between exposure of interest and outcome were explored with uni- and multivariable logistic regression analysis. The figure was made with modified art from Servier Medical Art (https://smart.servier.com/), provided by Servier, licensed under a Creative Commons Attribution 3.0 unported license.

We used blood samples from kidney transplant recipients to measure FOXP3 splice variants. In blood, FOXP3 transcripts are predominantly produced by T regulatory cells (2, 17, 18), which can be appreciated by its high tau score of 0.94 according to proteinatlas.org (www.proteinatlas.org/ENSG00000049768-FOXP3/immune+cell). Because FOXP3 expression and T regulatory cell counts decrease shortly after transplantation and then slowly increase (19–22), we used samples collected the first day after transplantation (within 1-4 days post-transplant) and 29 days after transplantation (within 26-32 days post-transplant). The study timeline and methodology are illustrated in **Figure 1**.

For *ad hoc* analysis, we were interested in the change of FOXP3 splice variants from pre-transplant to post-transplant levels. Therefore, we measured FOXP3 splice variant levels in available pre-transplant samples of included kidney transplant recipients. Available pre-transplant samples were collected within a week of transplantation.

Clinical data was collected through review of electronic medical records, e.g.: recipient age; sex; height; weight; comorbidities including diabetes mellitus and coronary artery disease; active tobacco use; cause of end-stage kidney disease; duration of dialysis (vintage) in months; type of dialysis (hemodialysis, peritoneal dialysis); number of transplantations; donor type (ABO-compatible, AB0-incompatible living donor, deceased donor); total number of human leukocyte antigen (HLA) mismatches; delayed graft function defined as the need for renal replacement therapy within the first week post-transplant; immunosuppressive induction therapy including IL2-receptor antibodies (Basiliximab), Thymoglobulin, corticosteroids and anti-CD20 antibodies (Rituximab); immunosuppressive maintenance therapy including tacrolimus, cyclosporine and mycophenolate acid. Donor data were obtained from medical records. For each participant, laboratory data and diagnose codes were extracted from the day of transplantation, until 365 days after transplantation.

### 2.3 Study population

MoMoTx participants who were recruited from the inception of MoMoTx until 10. August 2021 were screened for inclusion (N = 617). Patients who had available laboratory data for calculation of the outcome variable were included in the final analysis (N = 333). Pre-transplant samples were available in 150 of the included study participants.

### 2.4 Decline of eGFR as the outcome variable

We were interested in distinguishing kidney transplant recipients with stable allograft function from recipients with declining allograft function. Plasma creatinine levels were used to calculate estimated glomerular filtration rate (eGFR) using the Chronic Kidney Disease Epidemiology Collaboration equation which has recently been used in kidney transplant recipients (23–25). We calculated an eGFR slope between the highest eGFR values within a baseline and a follow-up period. Both periods spanned 28 days to account for potential temporary changes in eGFR. The baseline period was defined as 76 to 104 days post-transplant, and the follow-up period was defined as 337 to 365 days post-transplant. Finally, recipients who showed a decline of at least 5 ml/min/1.73m^2^ from baseline to follow-up eGFR were defined as recipients with declining eGFR. Recipients who did not show such a decline were defined as recipients with stable eGFR.

### 2.5 Determination of FOXP3 mRNA splice variants as the exposure variables of interest

**Figure 2:**
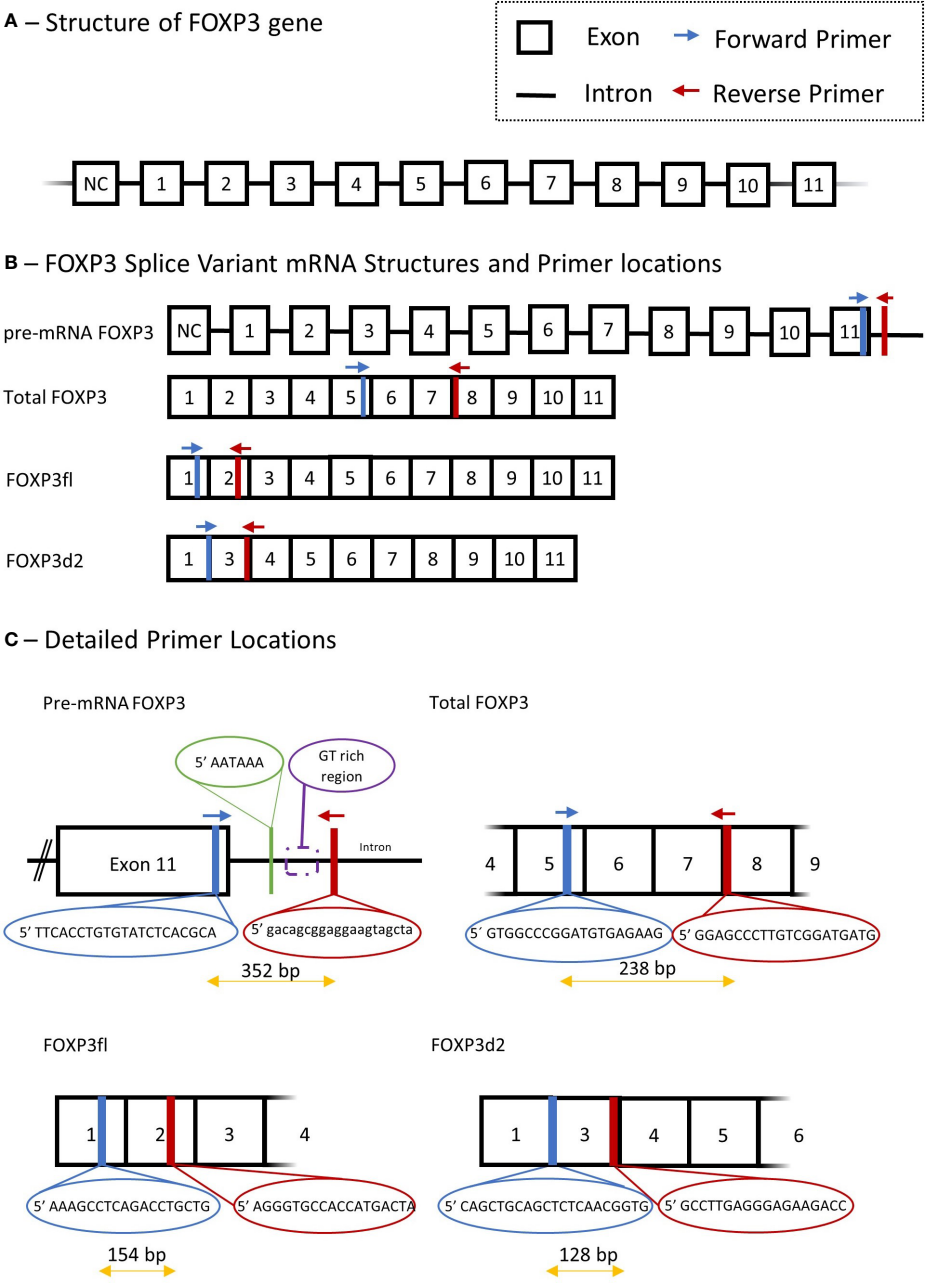
Schematic presentation of Forkhead Box P3 (FOXP3) gene, mRNA splice variant mRNA and detailed description of primers used in quantitative polymerase chain reaction. **(A)** structure of FOXP3 gene. **(B)** primer locations for different FOXP3 splice variants, Total FOXP3 detects both FOXP3fl and FOXP3d2. **(C)** Detailed primer locations and primer pair forward and reverse sequences. NC, non-coding exon; Bp, base pair.

FOXP3 mRNA splice variants were measured in peripheral blood mononuclear cells using quantitative reverse transcription polymerase chain reactions. Peripheral blood mononuclear cells were isolated from blood samples by density gradient centrifugation using Histopaque (Sigma-Aldrich, St. Louis, MO, USA; density 1.077 g/mL), the cell interphase was washed by centrifugation in Hanks’ balanced salt solution (Thermo Fisher scientific, Waltham, MA, USA), and suspended in TRIzol (Invitrogen, Thermo Fisher Scientific, Waltham, MA, USA). Total RNA was isolated using RNeasy Mini kit including RNase-free DNase set (Qiagen, Hilden, Germany). Complementary DNA was synthesized from 300 ng of total RNA by using a QuantiTect Reverse Transcription kit (Qiagen). Genomic DNA was eliminated by incubation of each RNA sample with the genomic DNA elimination mix for 4 minutes at 42°C followed by incubation with the manufacturer mix of quantiTect RT primer, reverse transcriptase, and reverse transcription buffer for 60 minutes at 37°C, followed by heating to 95° for 5 minutes. Finally, complementary DNA was used in SYBR green quantitative reverse transcription polymerase chain reaction, LightCycler 96 (Roche Diagnostics, Basel, Switzerland). The FOXP3 gene, FOXP3 mRNA splice variants and primer sets are illustrated in **Figure 2**.

We measured three FOXP3 splice variants with the following primer sets: Total mature RNA FOXP3 (Total FOXP3) which accounts for the two major variants of FOXP3 in human T regulatory cells (9), namely FOXP3fl and FOXP3d2; Pre-mature mRNA FOXP3 (pre-mRNA FOXP3) which accounts for FOXP3 mRNA that has not undergone RNA splicing, and thus contains intron and exon RNA; FOXP3 that lack exon 2 (FOXP3d2) which accounts for spliced FOXP3 mRNA where exon 2 has been skipped (9); Full length FOXP3 (FOXP3fl) which accounts for spliced FOXP3 mRNA in which exon skipping has not occurred, and all coding exons are present (9).

The quantitative reverse transcription polymerase chain reaction method was adapted from an already published method by our group (26). Quantitative reverse transcription polymerase chain reaction was performed using a solution containing 10 µL Fast Start Essential DNA Green Master mix (Roche Diagnostics, Denmark), 4 µL H_2_O and 2 µL of forward and reverse primer. For each sample, a mixture of solution and complementary DNA was added to a final volume of 20 µL. 15 µL of this solution was added with 5 µL of complementary DNA to measure Total FOXP3 and pre-mRNA FOXP3, while 18 µL was added to 2 µL of complementary DNA to measure FOXP3d2 and FOXP3fl. The reverse transcription settings were performed as follows: pre-incubation at 95°C for 10 minutes, 55 cycles with denaturation at 95°C for 10 seconds, annealing at 63°C for 10 seconds and extension at 72°C for 10 seconds. Quantification cycle values (Cq) for each reaction were determined using LightCycler 96 Software 1.1 (Roche Diagnostics, Denmark). We calculated a normalized target splice variant expression relative to β-actin with the following equation: Normalized ratio = ET^(CqR-CqT)^ with ET, efficiency of target amplification; CqT and CqR, quantification cycle at target/reference detection (26). The sizes of polymerase chain reaction products were 238 bp for Total FOXP3, 352 bp for pre-mRNA FOXP3, 128 bp for FOXP3d2 and 154 bp for FOXP3fl. Details of all primers, annealing temperature and efficiency are provided in **Supplementary Table 1**.

Repeated measures of normalized values of the splice variants did not differ when using 5 µL or 2 µL cDNA (**Supplementary Table 2**). We performed gel electrophoresis of the quantitative polymerase chain reaction product (**Supplementary Figure 1**) to ensure that each primer pair produced only one product. We also ensured that each sample had one melting peak in quantitative polymerase chain reaction analysis (**Supplementary Figure 2**). Finally, we observed low Inter- and intra-sample variability with repeated measures (**Supplementary Tables 3**, **4**). The treating physicians were unaware of the FOXP3 results.

We developed several primer pairs to measure the FOXP3 mRNA variant that lacks exon 2 and 7 (**Supplementary Table 5**). This FOXP3 splice variant accounts for less than 3% of FOXP3 transcripts in human T regulatory cells (9), and its expression may even be lower in kidney transplant recipients because immunosuppressive therapy may decrease T regulatory cell abundance and FOXP3 expression (6). The low abundance could be observed using several primer pairs, as these produced high Cq values, irregular melting peaks, and gel electrophoresis did not show a specific primer product in 55% of samples (**Supplementary Figure 3**). Thus, the low abundance of the FOXP3 variant lacking exons 2 and 7 in peripheral blood mononuclear cells hinders its accurate measurement with quantitative polymerase chain reaction (27). Furthermore, the FOXP3 variant lacking both exon 2 and exon 7 is not involved in T regulatory cell differentiation, lineage stability or establishment of suppressive function (9). Therefore, we measured the two most abundant FOXP3 splice variants, FOXP3d2 and FOXP3fl, which is in agreement with the literature (27, 28).

### 2.6 Other variables

Danish ICD-10 codes were used to identify patients that experienced rejection episodes (**Supplementary Table 6**). Those who had at least one of the specified codes were coded as having at least 1 rejection episode within the first post-transplant year.

### 2.7 Statistical analysis

Continuous variables were reported as median [interquartile range] and compared using two-sided Wilcoxon signed rank test or Wilcoxon rank sum test as appropriate, unless otherwise specified. Categorical data are reported as number (percent) and compared using Fisher’s exact or chi-squared-test as appropriate. A two-sided p-value less than 0.05 was considered statistically significant.

We used logistic regression to test the association between the FOXP3 splice variants as the exposure variables of interest and declining eGFR as the outcome variable. The association was tested both in uni- and multivariable logistic regression. Results of logistic regression are reported as odds ratio (OR) and 95% confidence interval (95% CI). FOXP3 splice variant values were converted to a logarithmic scale to simplify interpretation of results including OR. Missing values (**Supplementary Table 7**) were not missing completely at random (Little’s test p < 0.01) and were therefore not imputed.

**Figure 3:**
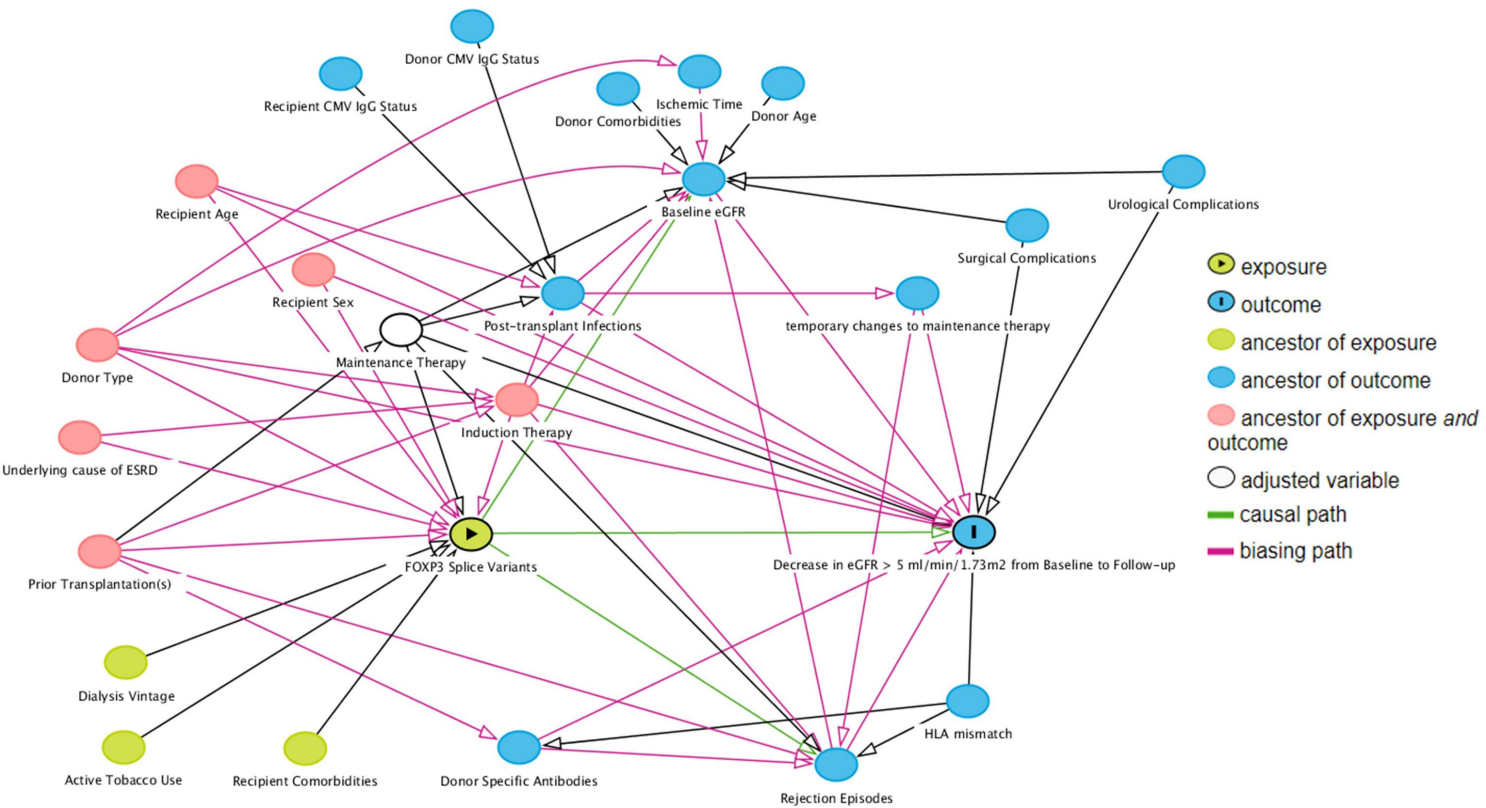
Direct acyclic graph of the exposure variable of interest (exposure), outcome and their ancestor variables. Using the backdoor pathway criteria, ancestors of exposure and outcome variables (red) were considered potential confounding factors and included in multivariable logistic regression analysis. Maintenance therapy is adjusted by design – all included patients received tacrolimus with specified blood targets, and a uniform dose of mycophenolate acid. Causal paths are based on the literature and design following clinical practices. Baseline estimated glomerular filtration rate (eGFR) was defined as the highest eGFR within 76 to 104 days post-transplant, and follow-up eGFR was the highest eGFR within 337 to 365 days post-transplant.

To choose covariates for the multivariable analysis, we used directed acyclic graph with back-door pathway criteria (29, 30), which were simulated with the online tool DAGitty (www.dagitty.net/dags.html) (**Figure 3**). Common ancestors of the exposure variable of interest and the outcome were considered potential confounding factors and included in the multivariable analysis. The assumption in the generated directed acyclic graph were based on the literature, all assumptions are cited and detailed in **Supplementary Table 8**.

We used likelihood ratio test to test the association of the exposure in logistic regression. To do this, we defined the full model including declining eGFR as the outcome variable, a FOXP3 splice variant as the exposure variable of interest and confounding factors as co-variates. The nested model consisted of the full model excluding the exposure variable of interest. Next, the goodness of fit was tested with Stukel’s test. All statistical analyses were carried out using R (version 4.2.2, R Foundation for Statistical Computing, Vienna, Austria).

## 3 Results

### 3.1 Study participant characteristics

**Figure 4:**
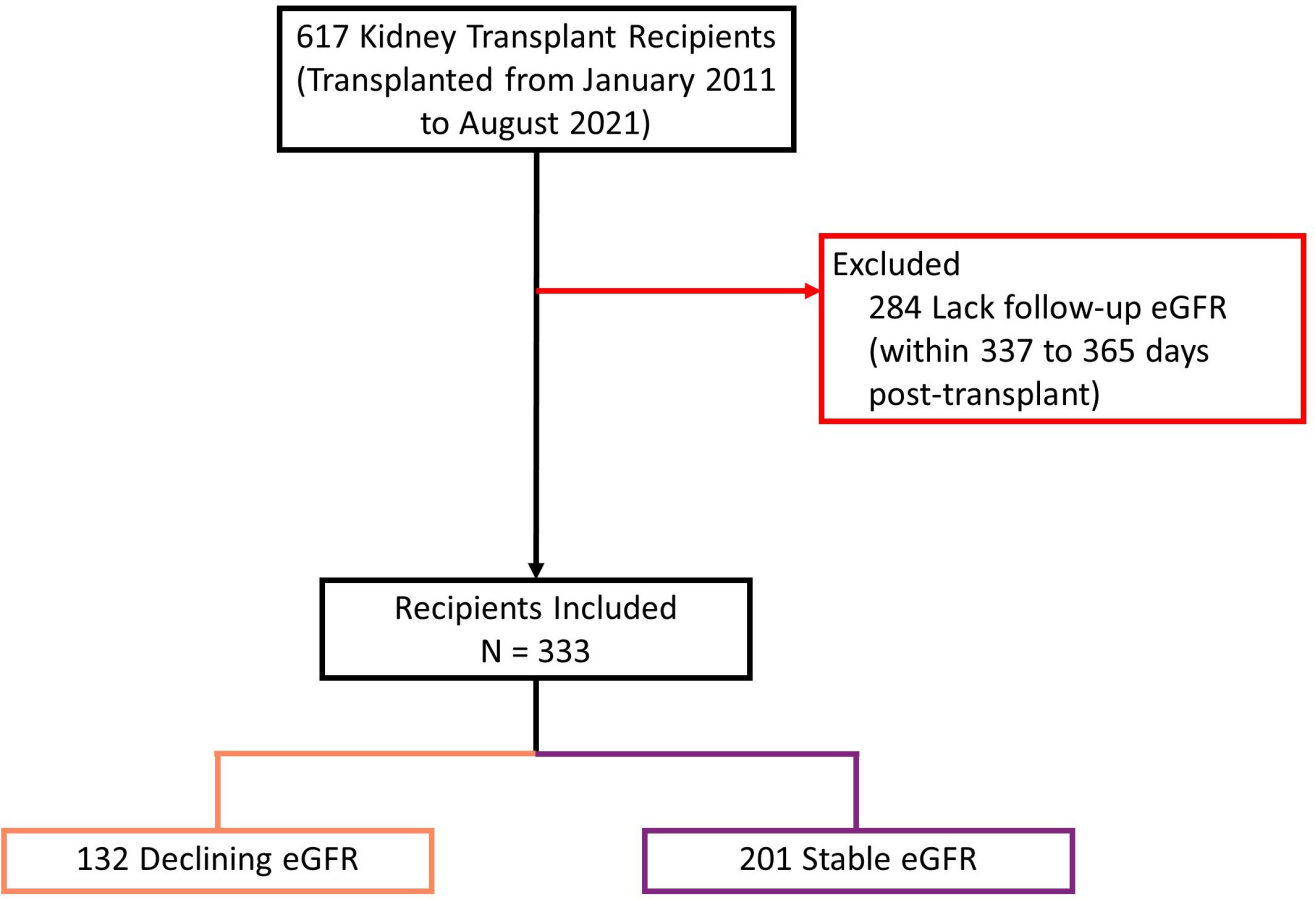
Study population flow diagram describing inclusion process. eGFR: estimated glomerular filtration rate. Declining eGFR: a decrease from baseline (76 to 104 days post-transplant) to follow-up (337 to 365 days post-transplant) eGFR above 5 ml/min/1.73m^2^.

We screened 617 kidney transplant recipients, and 333 recipients were included in the final analysis (**Figure 4**). In the excluded 284 recipients, laboratory data was not available at baseline, follow-up or both, and the outcome variable could not be calculated. Clinical and biochemical characteristics were not significantly different between included and excluded recipients (**Supplementary Tables 9**, **10**). Of the included recipients, 216 (65%) were male and the median age was 54 years [IQR 43 to 63]. Barring differences in eGFR, recipients did not differ in baseline characteristics (**Table 1**).

**Table 1:** Peri-, and post-transplant characteristics for study population by presence or absence of declining eGFR.

Characteristic	All patients (N = 333)	Stable eGFR (N = 201)	Declining eGFR (N= 132)	P-value
Recipient Characteristics				
Age (years)	54 [43 to 63]	54 [43 to 62]	52 [42 to 63]	0.80^a^
Male sex, N (%)	216 (65%)	123 (61%)	93 (70%)	0.08^c^
Height (cm)	174 [167 to 182]	175 [168 to 181]	174 [166 to 182]	0.70 ^a^
Weight (kg)	81 [70 to 93]	81 [69 to 93]	82 [71 to 93]	0.60 ^a^
Diabetes, N (%)	67 (20%)	38 (19%)	29 (22%)	0.50 ^c^
Coronary artery disease, N (%)	33 (9.9%)	22 (11%)	11 (8.3%)	0.30 ^c^
Active tobacco use, N (%)	96 (29%)	57 (28%)	39 (30%)	0.80 ^c^
Cause of kidney disease, N (%) Glomerulonephritis Diabetic nephropathy Hypertensive nephropathy Other Unknown	106 (31%) 54 (16%) 42 (13%) 72 (22%) 59 (18%)	63 (32%) 31 (15%) 20 (10%) 51 (25%) 36 (18%)	43 (33%) 23 (17%) 22 (17%) 21 (16%) 23 (17%)	0.20 ^b^
Duration of dialysis (months)	12 [2 to 30]	12 [2 to 26]	12 [2 to 33]	0.40 ^a^
Type of dialysis, N (%) Pre-emptive Hemodialysis Peritoneal dialysis	75 (22%) 181 (54%) 77 (23%)	46 (23%) 108 (54%) 47 (23%)	29 (22%) 73 (55%) 30 (23%)	0.90^b^
Transplantation, N (%) First transplant Second/more	283 (85%) 50 (15%)	172 (86%) 29 (14%)	111 (84%) 21 (16%)	0.70^c^
Donor type, N (%) Deceased Living ABO-compatible Living ABO-incompatible	191 (57%) 99 (30%) 43 (13%)	110 (55%) 67 (33%) 24 (12%)	81 (61%) 32 (25%) 19 (14%)	0.20 ^b^
Number of HLA mismatches (range within 0-8)	3 [2 to 4]	3 [2 to 4]	3 [2 to 4]	0.10 ^a^
Plasma creatinine pre-transplant (µmol/l)	710 [552 to 890]	689 [536 to 833]	713 [570 to 916]	0.20 ^a^
Delayed graft function	47 (14%)	31 (15%)	16 (12%)	0.40 ^c^
Baseline eGFR	55 [42 to 67]	52 [39 to 63]	59 [48 to 74]	< 0.01 ^a^
Follow-up eGFR	52 [36 to 64]	57 [41 to 70]	46 [31 to 59]	< 0.01 ^a^
Experienced at least 1 rejection episode within the first-year post-transplant	70 (21%)	36 (18%)	34 (26%)	0.08 ^c^
Induction and Maintenance Therapy				
IL2-receptor antibodies (*Basiliximab*), N (%)	272 (82%)	170 (85%)	102 (77%)	0.10 ^c^
Thymoglobulin, N (%)	52 (16%)	27 (13%)	25 (19%)	0.20 ^c^
Corticosteroids, N (%)	92 (28%)	53 (26%)	39 (30%)	0.50 ^c^
Anti-CD20 antibodies (*Rituximab)*, N (%)	69 (21%)	39 (19%)	30 (23%)	0.40 ^c^
Maintenance therapy, N (%) Tacrolimus Cyclosporine Mycophenolate Acid	333 (100%) 0 333 (100%)	201 (100%) 0 201 (100%)	132 (100%) 0 132 (100%)	–
Donor Characteristics				
Age (years)	55 [46 to 64] (Missing = 85)	56 [47 to 64] (Missing = 46)	54 [45 to 65] (Missing = 39)	0.70 ^a^
Male sex, N (%)	107 (42%) (Missing = 81)	69 (44%) (Missing = 45)	38 (40%) (Missing = 36)	0.50 ^c^
Cold ischemic time (minutes)	780 [585 to 1080] (Missing = 156)	780 [578 to 1080] (Missing = 98)	780 [600 to 1022] (Missing = 58)	0.80 ^a^

Stable eGFR: a difference between baseline (highest eGFR value within 76 to 104 days post-transplant) and follow-up (highest eGFR value within 337 to 365 days post-transplant) eGFR < 5 ml/min/1.73m^2^. Declining eGFR: a decline from baseline eGFR to follow-up eGFR of at least 5 ml/min/1.73m^2^. eGFR: estimated glomerular filtration rate, calculated using serum creatinine with the CKD-EPI formula. Other causes of end stage kidney disease encompass hydronephrosis, cancer and polycystic kidney disease. HLA: human leukocyte antigen. Numerical data is presented as median [interquartile range] and tested with Wilcoxon rank sum test (a), categorical data is presented as number (percent) and tested with Pearson’s Chi squared test (b) or Fischer’s exact test (c) as appropriate.

### 3.2 The outcome variable distinguishes between recipients with stable - and declining eGFR

**Figure 5:**
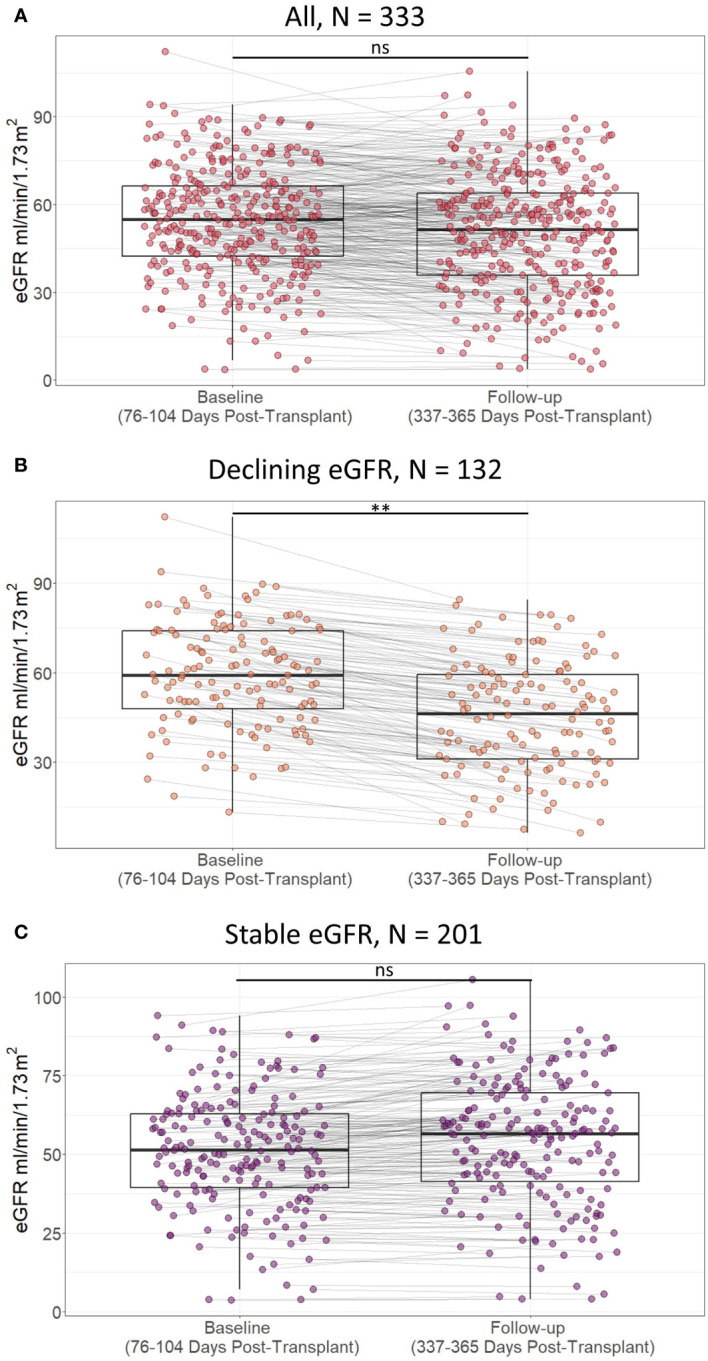
Scatterplot of estimated glomerular filtration rate (eGFR) in included patients at baseline (76 to 104 days post-transplant) and follow-up (337 to 365 days post-transplant) according to presence of decline in eGFR within the first post-transplant year in **A**) all included study participants, **B**) study partcipants with declining estimated glomerular filtration rate (eGFR), and **C**) study participants with stable eGFR. Lines indicate statistical comparison with Wilcoxon signed rank test. ns, not significant; **, p<0.01. Declining eGFR: a decrease from baseline (76 to 104 days post-transplant) to follow-up (337 to 365 days post-transplant) eGFR above 5 ml/min/1.73m2. Stable eGFR: the difference between baseline and follow-up eGFR is below 5 ml/ min/1.73m2.

For all included kidney transplant recipients, the median baseline eGFR was 55 ml/min/1.73m^2^ [IQR 42 to 67], and the median follow-up eGFR was 52 ml/min/1.73m^2^ [IQR 36 to 64]. 132 recipients had a decline of at least 5 ml/min/1.73m^2^ from baseline to follow-up eGFR, while 201 recipients had stable eGFR values (**Figure 5**). The difference between baseline and follow-up eGFR was larger in recipients with declining eGFR than recipients with stable eGFR, median change in eGFR 11 (ml/min/1.73m^2^) [IQR 8 to 16] vs -2 (ml/min/1.73m^2^) [IQR -6 to 1], p < 0.01. Follow-up eGFR was not significantly lower than baseline in recipients with stable eGFR, follow-up eGFR 57 ml/min/1.73m^2^ [IQR 41 to 70] vs baseline eGFR 52 ml/min/1.73m^2^ [IQR 39 to 63] one sided Wilcoxon signed rank test p = 1.00. Thus, the outcome definition provides a distinction between kidney transplant recipients with a larger tendency of decline in eGFR and recipients who have stable eGFR values at baseline and follow-up.

### 3.3 Association of low day one FOXP3fl mRNA splice variant with declining eGFR

**Figure 6:**
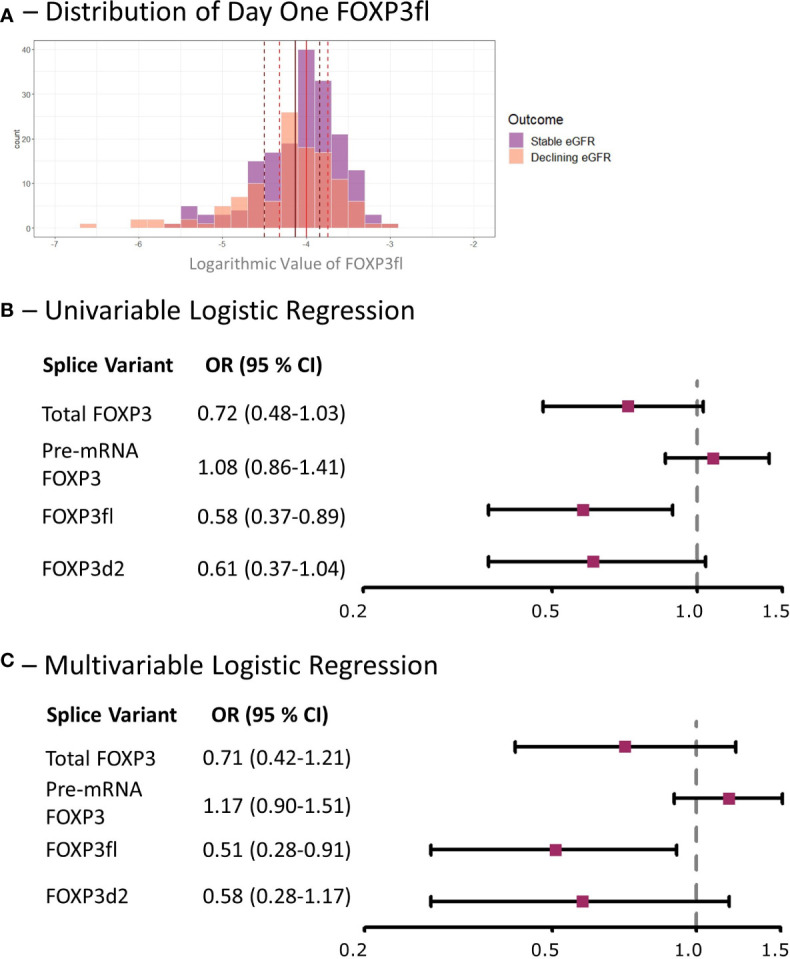
Low day one FOXP3fl is associated with a decline in eGFR within the first post-transplant year in kidney transplant recipients. **(A)** Distribution of logarithmic values of FOXP3fl normalized to β-actin, vertical lines indicate medians and dotted lines indicate interquartile range (black represents declining eGFR and red represents stable eGFR), there was a statistically significant difference between medians (Wilcoxon rank sum test, p = 0.02). **(B)** Univariable logistic regression analysis results where FOXP3 spice variants measured one day post-transplant are the exposure variables of interest and a declining estimated glomerular filtration rate (eGFR) within the first-year post-transplant is the outcome. **(C)** Multivariable Logistic regression analysis results where the exposure variable of interest is FOXP3 splice variants measured one day post-transplant, a declining estimated glomerular filtration rate (eGFR) within the first-year post-transplant is the outcome, and the adjusted co-variates are: recipient age, recipient sex, donor type, prior transplantation, underlying cause of kidney disease and immunosuppressive induction therapy. Declining eGFR: a decrease from baseline (76 to 104 days post-transplant) to follow-up (337 to 365 days post-transplant) eGFR above 5 ml/min/1.73m^2^. Stable eGFR: the difference between baseline and follow-up eGFR is below 5 ml/min/1.73m^2^. OR, odds ratio; CI, confidence interval.

Day one FOXP3fl splice variant levels were significantly lower in recipients with declining eGFR compared to recipients with stable eGFR, logarithmic value -4.13 [IQR -4.50 to -3.84] vs -4.00 [-4.32 to -3.74], p = 0.02 (**Table 2**, **Figure 6A**, **Supplementary Figure 4**). In univariable analysis (**Table 3**, **Figure 6B**), there was a statistically significant association between low levels of day one FOXP3fl and declining eGFR OR 0.58 (95% CI 0.37-0.89: p = 0.01).

**Table 2:** Forkhead Box P3 (FOXP3) splice variants in all patients, and according to stable or declining estimated glomerular filtration rate (eGFR).

Days post-transplant	Variant	All (N = 333)	Stable eGFR (N = 201)	Declining eGFR (N = 132)	P-value
One	Total FOXP3	-3.72 [-4.00 to -3.45]	-3.70 [-3.97 to -3.42]	-3.73 [-4.07 to -3.50]	0.30
	Pre-mRNA FOXP3	-4.40 [-4.65 to -4.06]	-4.41 [-4.69 to -4.11]	-4.40 [-4.64 to -3.91]	0.30
	FOXP3fl	-4.04 [-4.37 to -3.77]	-4.00 [-4.32 to -3.74]	-4.13 [-4.50 to -3.84]	0.02
	FOXP3d2	-3.78 [-4.04 to -3.53]	-3.73 [-4.01 to -3.50]	-3.88 [-4.13 to -3.56]	0.06
29	Total FOXP3	-3.62 [-3.91 to -3.36]	-3.63 [-3.91 to -3.41]	-3.61 [-3.92 to -3.30]	0.80
	Pre-mRNA FOXP3	-4.37 [-4.64 to -4.11]	-4.41 [-4.72 to -4.13]	-4.30 [-4.60 to -4.03]	0.11
	FOXP3fl	-3.89 [-4.22 to -3.64]	-3.89 [-4.22 to -3.65]	-3.86 [-4.27 to -3.64]	0.90
	FOXP3d2	-3.70 [-3.97 to -3.45]	-3.70 [-3.95 to -3.45]	-3.70 [-4.03 to -3.45]	0.60

Data are logarithmic values of FOXP3 splice variants normalized to β-actin. Stable eGFR: a difference between baseline (highest eGFR value within 76 to 104 days post-transplant) and follow-up (highest eGFR value within 337 to 365 days post-transplant) eGFR < 5 ml/min/1.73m^2^. Declining eGFR: a decline from baseline eGFR to follow-up eGFR of at least 5 ml/min/1.73m^2^. Numerical data is presented as median [interquartile range) and tested with Wilcoxon rank sum test.

**Table 3:** Univariable and multivariable logistic regression analysis exploring potential association between logarithmic values of FOXP3 splice variants and decline in estimated glomerular filtration rate of at least 5 ml/min/1.73m^2^ from baseline (highest eGFR value within 76 to 104 days post-transplant) until follow-up (highest eGFR value 337-365 days post-transplant).

Time-point of FOXP3 splice variant measurement (days after transplantation)	FOXP3 splice variant	Univariable analysis		Multivariable analysis	
		Odds ratio (95% confidence interval)	P-value	Odds ratio (95% confidence interval)	P-value
One	Total FOXP3	0.72 (0.48-1.03)	0.07	0.71 (0.42-1.21)	0.21
	FOXP3 pre-mRNA	1.08 (0.86-1.41)	0.50	1.17 (0.90-1.51)	0.23
	FOXP3fl	0.58 (0.37-0.89)	0.01	0.51 (0.28-0.91)	0.02
	FOXP3d2	0.61 (0.37-1.04)	0.05	0.58 (0.28-1.17)	0.13
29	Total FOXP3	0.97 (0.64-1.46)	0.89	1.18 (0.71-1.98)	0.50
	FOXP3 pre-mRNA	1.53 (1.03-2.31)	0.04	1.59 (1.03-2.48)	0.03
	FOXP3fl	0.91 (0.53-1.56)	0.74	1.17 (0.58-2.35)	0.64
	FOXP3d2	0.72 (0.39-1.32)	0.29	0.87 (0.39-1.96)	0.74

Included covariates in multivariable are recipient age, recipient male sex, donor type, first vs. prior kidney transplantations, cause of kidney disease, use of IL2-receptor antibodies (Basiliximab), use of thymoglobulin and use of corticosteroids induction therapy.

**Figure 7:**
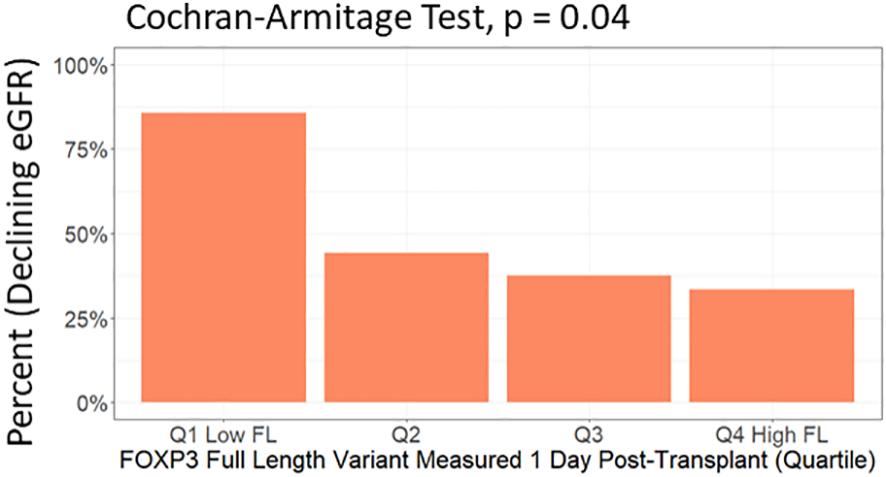
Test of trend between day one FOXP3fl levels in quartiles and percent of included patients who experienced a decline in estimated glomerular filtration rate (eGFR) within the first post-transplant year. FOXP3fl: mature FOXP3 mRNA containing all exons. Declining eGFR: a decrease from baseline (76 to 104 days post-transplant) to follow-up (337 to 365 days post-transplant) eGFR above 5 ml/min/1.73m^2^.

In multivariable analysis (**Table 3**, **Figure 6C**), we adjusted for the covariates: recipient age and sex, donor type, first - vs. re-transplantation, cause of kidney disease, use of basiliximab, use of thymoglobulin and use of corticosteroid induction therapy. The association between low levels of day one FOXP3fl and declining eGFR remained statistically significant, OR 0.51 (95% CI 0.28-0.91: p = 0.02). Additionally, there was no evidence of model deficiency (Stukel’s test p = 0.69), and the model was appropriate (likelihood ratio test p = 0.02) (**Supplementary Table 11**). When stratifying FOXP3fl into quartiles, we observed a statistically significant trend (Cochran-Armitage test p = 0.04) where those with lower day one FOXP3fl had higher rates of declining eGFR (**Figure 7**).

In contrast, we did not observe an association of other day one FOXP3 splice variant levels with declining eGFR, total FOXP3 OR 0.71 (95% CI 0.42-1.21: p = 0.21), pre-mRNA FOXP3 OR 1.17 (95% CI 0.90-1.51: p = 0.23), and FOXP3d2 OR 0.58 (95% CI 0.28-1.17: p = 0.50). Associations of remaining covariates are displayed in **Supplementary Tables 12**, **13**.

### 3.4 Association of high day 29 pre-mRNA FOXP3 splice variant with declining eGFR

Comparing day 29 FOXP3 splice variant levels in recipients with declining eGFR and recipients with stable eGFR, we found that the levels were statistically indistinguishable (**Table 2**, **Supplementary Figures 4**, **5A**). In univariable analysis (**Supplementary Figure 5B**) high levels of day 29 pre-mRNA FOXP3 were associated with declining eGFR. In contrast, day 29 total FOXP3, FOXP3fl and FOXP3d2 did not show an association with declining eGFR. We observed similar results in multivariable analysis (**Supplementary Figure 5C**). High levels of day 29 pre-mRNA FOXP3 remained significantly associated with declining eGFR OR 1.59 (95% CI 1.03-2.48: p = 0.02). When stratified into quartiles, the trend between higher pre-mRNA FOXP3 levels and declining eGFR was P = 0.08 by Cochran-Armitage test (**Supplementary Figure 5D**). Day 29 total FOXP3 OR 1.18 (95% CI 0.71-1.98: p = 0.50), - FOXP3fl OR 1.17 (95% CI 0.58-2.35: p = 0.64) and - FOXP3d2 OR 0.87 (95% CI 0.39-1.96: p = 0.74), were not significantly associated with declining eGFR.

### 3.5 Changes in FOXP3 Splice variant levels are most prominent in recipients with declining eGFR

**Figure 8:**
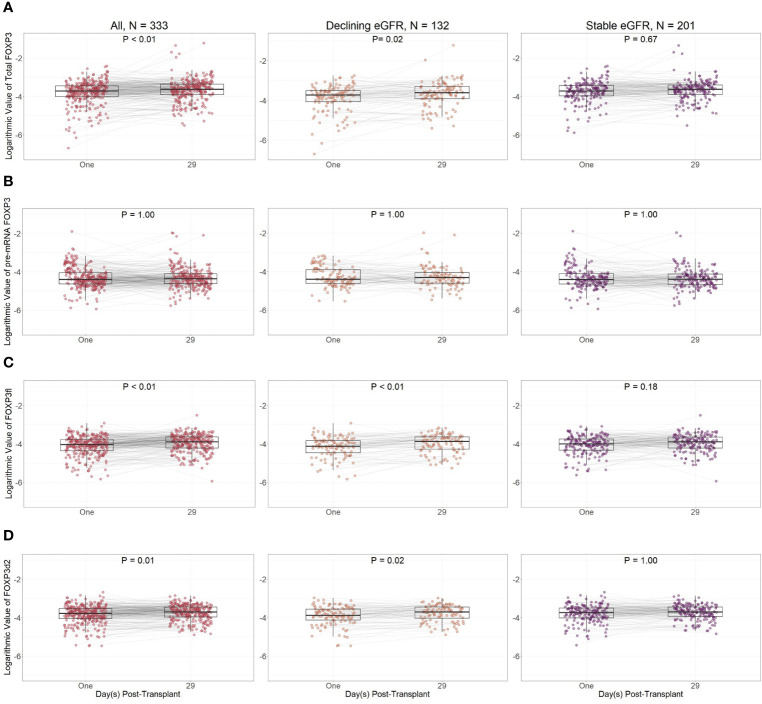
Logarithmic values of FOXP3 splice variants in included patients according to post-transplant day. **(A)** Total FOXP3: all mature splice variants, **(B)** pre-mRNA FOXP3, **(C)** FOXP3fl: mature FOXP3 mRNA containing all exons, **(D)** FOXP3d2: mature FOXP3 mRNA in which exon 2 is spliced. Comparisons were performed with Bonferroni adjusted Wilcoxon signed rank test. Declining eGFR: a decrease from baseline (76 to 104 days post-transplant) to follow-up (337 to 365 days post-transplant) eGFR above 5 ml/min/1.73m^2^. Stable eGFR: the difference between baseline and follow-up eGFR is below 5 ml/min/1.73m^2^.

Except for pre-mRNA FOXP3, FOXP3 splice variant levels were lower at day one compared to day 29 in all kidney transplant recipients (**Figure 8**). However, in sub-group analysis, the difference between FOXP3 splice variants measured at day one and 29 was more prominent in recipients with declining eGFR.

When comparing day one and day 29 FOXP3 splice variant levels in recipients with declining eGFR, day one FOXP3 splice variants were significantly lower when measured as total FOXP3 (p = 0.02), FOXP3d2 (p = 0.02), and FOXP3fl (p<0.01). Pre-mRNA FOXP3 levels did not change p = 1.00.

In recipients with stable eGFR, FOXP3 splice variant levels did not change between day one and day 29. We did not observe a change in total FOXP3 (p = 0.67), pre-mRNA FOXP3 (p = 1.00), FOXP3d2 (p = 1.00) or FOXP3fl (p = 0.18).

In *ad hoc* analysis, pre-transplant samples were available in 53 (35%) recipients with declining eGFR and 97 (75%) with stable eGFR (**Table 4**). Pre-Transplant FOXP3fl splice variant levels did not differ between recipients with stable eGFR and recipients with declining eGFR (p = 0.74). However, the fall in day one FOXP3fl levels was larger in recipients with declining eGFR compared to recipients with stable eGFR (p = 0.03) (**Supplementary Figure 6**).

**Table 4:** Logarithmic values of FOXP3 splice variant levels normalized to β-actin according to variant, time-point of measurement and group. Data are represented as median [interquartile range].

Declining eGFR						
Variable	Pre-transplant levels	Levels one day post-transplant	Levels 29 days post-transplant	Comparison of pre-transplant vs day one samples	Comparison of pre-transplant levels vs day 29 levels	Comparison of day one vs day 29 levels
**Total FOXP3**	-3.35 [-3.52 to -3.09]	-3.72 [-4.13 to -3.50]	-3.59 [-3.89 to -3.27]	< 0.01	< 0.01	0.58
**Pre-mRNA**	-4.32 [-4.50 to -4.04]	-4.40 [-4.64 to -4.10]	-4.30 [-4.53 to -4.06]	1.00	1.00	1.00
**FOXP3fl**	-3.63 [-3.90 to -3.40]	-4.14 [-4.52 to -3.89]	-3.85 [-4.28 to -3.67]	< 0.01	< 0.01	< 0.01
**FOXP3d2**	-3.44 [-3.63 to -3.34]	-3.88 [-4.08 to -3.62]	-3.66 [-3.96 to -3.45]	< 0.01	0.02	0.03

Stable eGFR						
Variable	Pre-transplant levels	Levels one day post-transplant	Levels 29 days post-transplant	Comparison of pre-transplant vs day one samples	Comparison of pre-transplant levels vs day 29 levels	Comparison of day one vs day 29 levels
**Total FOXP3**	-3.33 [-3.53 to -3.11]	-3.61 [-3.96 to -3.41]	-3.63 [-4.02 to -3.37]	< 0.01	< 0.01	1.00
**Pre-mRNA**	-4.31 [-4.48 to -4.12]	-4.46 [-4.68 to -4.13]	-4.41 [-4.72 to -4.20]	0.19	0.80	1.00
**FOXP3fl**	-3.63 [-3.86 to -3.41]	-3.93 [-4.25 to -3.71]	-3.90 [-4.22 to -3.62]	< 0.01	< 0.01	1.00
**FOXP3d2**	-3.48 [-3.60 to -3.32]	-3.70 [-3.98 to -3.48]	-3.73 [-3.95 to -3.49]	< 0.01	< 0.01	1.00

Comparisons were performed with Wilcoxon signed-rank test and Bonferroni adjusted. eGFR: estimated glomerular filtration rate. Declining eGFR: a decrease from baseline eGFR (highest eGFR within 76 to 104 days post-transplant) to follow-up eGFR (highest eGFR within 337 to 365 days post-transplant) of at least 5 ml/min/1.73m^2^. Stable eGFR: the difference between baseline - follow-up eGFR is < 5 ml/min/1.73m^2^.

Taken together, our peripheral blood sample based measurement of FOXP3 splice variant levels allow early identification of kidney transplant recipients with declining eGFR, as these are characterized by low FOXP3fl splice variant levels despite comparable pre-transplant levels to recipients with stable eGFR.

## 4 Discussion

The present results show that, measured on the first postoperative day, reductions in FOXP3 full length splice variant levels in kidney transplant recipients are associated with a decline in eGFR (>5 ml/min/1.73m^2^) in the first-year post-transplant. even when adjusted for confounding factors. Such a decline in eGFR is a surrogate marker for long-term allograft survival (1).

FOXP3 transcripts are essential for T-regulatory cell differentiation, function, and stability (6, 9). Without FOXP3, T-regulatory cells fail to regulate tolerance to allo- and autoantigens (4, 5, 31). In kidney transplant recipients, allograft function and survival depend on such tolerance to alloantigens as allograft injury occurs in its absence (32). Therefore, we investigated FOXP3 expression as a marker of tolerance in kidney transplant recipients.

FOXP3 is predominantly produced by T-regulatory cells (2, 17). The two most common variants, FOXP3fl and FOXP3d2, differ in their expression of exon two (9). Exon two is skipped in FOXP3d2 and expressed in FOXP3fl; it partly codes for a nuclear export factor, which can facilitate relocation of FOXP3fl into the cytoplasm of T-regulatory cells, and partly for a repressor domain (12, 13). While the cytoplasmic actions of FOXP3fl are largely unclear (9), FOXP3fl has been shown to alter the transcription of several genes (14). The collective functions of FOXP3fl affect T-regulatory cell homeostasis and cell lineage maintenance and are essential as humans develop systemic autoimmune disorders when FOXP3fl transcription is impaired (11). According to recent findings, a decrease in FOXP3fl expression may shift the immune system toward a pro-inflammatory state by disrupting T-regulatory cell stability, homeostasis, and function (11).

Only a few studies have investigated the association between FOXP3 mRNA levels and kidney allograft tolerance. In a study with 86 kidney transplant recipients, Canossi et al. reported a positive correlation of FOXP3 mRNA levels in peripheral blood and one-year post-transplant allograft function <60 ml/min/1.73m^2^, but in their study FOXP3 was not associated with allograft function <60 ml/min/1.73m^2^ in multivariable analysis (33). In contrast, Iwase et al. reported an association of low FOXP3 mRNA in peripheral blood with biopsy proven chronic rejection (7). To date FOXP3 splice variants have not been investigated in kidney transplant recipients.

Changes in FOXP3 splice variant expression has been studied in autoimmune diseases (9, 11). Similar to our results, low FOXP3fl levels have been associated with pro-inflammatory conditions such as antineutrophil cytoplasmic antibody-associated vasculitis (34), and systemic lupus erythematosus (35). In contrast, other studies reported an association between higher FOXP3fl levels and pro-inflammatory conditions (27, 36, 37). This may be explained by the paradoxical increase in FOXP3 levels during active inflammation (36, 38–40). Thus, in absence of active inflammation, low FOXP3fl levels seem to be associated with a tendency to develop pro-inflammatory conditions.

Our results show that FOXP3 splice variant mRNA levels decrease following transplantation, and that such a decrease in FOXP3fl can characterize recipients who are less tolerant to the transplanted allograft. Following transplantation, FOXP3 levels can decrease due to immunosuppressive therapy (6). FOXP3 levels can also decrease in peripheral blood because the main producers of FOXP3 transcripts, T-regulatory cells, migrate to sites of active inflammation (41). Besides changes in FOXP3fl, clinical and biochemical data were similar in recipients with stable and declining eGFR. This includes factors that could illicit increased inflammation and subsequent T-regulatory cell migration to the allograft, such as HLA total mismatch, and cold ischemic time. These results do not support the notion that FOXP3fl levels decreased due to increased migration of T-regulatory cells to the kidney allograft because of an inflammatory response. Furthermore, the non-significant change of total FOXP3 between kidney transplant recipients with declining eGFR and stable eGFR also contradicts the explanation of T-regulatory cells to the kidney allograft because of an inflammatory response.

Immunosuppressive therapies such as thymoglobulin, basiliximab and calcineurin inhibitors, are intended to inhibit pro-inflammatory T effector cells, but they also affect T-regulatory cells and FOXP3 expression (6). Thymoglobulin decreases FOXP3 levels by depleting the absolute numbers of T-regulatory cells (19, 20, 42, 43). Basiliximab also decreases FOXP3 levels by inhibiting its expression through the interleukin 2 – signal transducer and activator of transcription 5B – FOXP3 pathway (6, 44, 45). This pathway is vital for T-regulatory cell development, stability, and function (44). Finally, calcineurin inhibitors decrease FOXP3 expression by disrupting the translocation of the nuclear factor of activated T cells into the T-regulatory cell nucleus (46, 47). The association between low FOXP3fl levels and declining eGFR might be explained by an unintended decrease in FOXP3 levels due to the action of immunosuppressive therapy. The resulting decrease of FOXP3fl might disrupt T-regulatory cell suppressive function, cell lineage stability, and homeostasis, which might shift the immune system into a pro-inflammatory state. Most importantly, our results demonstrate that changes in FOXP3 splice variant levels are not uniformly distributed in kidney transplant recipients thus being able to characterize recipients with a lower degree of allograft tolerance.

Our outcome definition has several strengths. A decline in eGFR has consistently been shown to be associated with end stage renal disease and mortality (1), and substantially reduce required sample size and follow-up time compared to other outcomes (48). We relied on the highest eGFR value within each period to account for the well-known assay variability of creatinine-based methods, that can result in eGFR slopes underestimating the severity of kidney function decline (49, 50). The baseline function was derived from values collected three months after transplantation, in analogy with earlier studies (51, 52). A sustained decline of 5 ml/min/1.73m^2^/year has clinical relevance (53), and has been used as an endpoint in multiple studies (54–57). Finally, one-year post-transplant decline of eGFR provides a better predictor of long-term graft survival and long term-eGFR than other outcomes (58).

The declining eGFR group had a higher percentage of male kidney transplant recipients compared to those with stable eGFR, although the difference was borderline significant (**Table 2**). Furthermore, male sex was the only co-variate that was significantly associated with declining eGFR in the models testing the associations of day one FOXP3 levels with declining eGFR (**Supplementary Table 12**). Male sex was however not associated with declining eGFR in the models testing the associations of day 29 FOXP3 levels with declining eGFR (**Supplementary Table 13**). Therefore, the data is contradictory and an association between male sex and declining eGFR is not clear in our dataset. Furthermore, strong evidence suggests that younger females are at higher risk of graft loss (59–61), which contradicts the results linking male sex to a sharper decline in kidney transplant function.

Our study has also some limitations. As an observational study, there is a risk of selection bias as some patients did not meet the inclusion criteria, but we confirmed that baseline characteristics were similar in excluded patients and included patients. There is also a risk of confounding, which we attempted to limit with directed acyclic graph driven multivariable analysis. However, residual confounding due to unknown confounders could impact the results. Furthermore, the participants were included from a single region in Denmark. Consequently, most recipients at our center are of European decent, warranting reproduction of our results in non-Europeans. Furthermore, the analysis would have benefitted from information on data such as the presence of pre-transplant donor specific antibodies, or histological diagnoses from any biopsies performed during rejection episodes.

In conclusion, lower FOXP3 full length splice variant levels measured in kidney transplant recipients on the first post-operative day were associated with a decline of eGFR of more than 5 ml/min/1.73m^2^ within the first-year post-transplant. FOXP3 full length splice variant transcript levels may indicate the balance between immunosuppression and allograft tolerance and may provide a promising tool to help guide personalized immunosuppressive treatment. With increasing interest in T-regulatory cell therapy in solid organ transplantation, FOXP3 splice variants may also provide insight into pathophysiological pathways and help guiding personalized screening post-transplant.

## Data availability statement

Data contains personal patient information and can be shared only after permission by the Danish Data Protection Agency. Further inquiries can be directed to the corresponding author/s.

## Ethics statement

The studies involving humans were approved by Den Videnskabsetiske Komite for Region Syddanmark, Project-ID: 20100098. The studies were conducted in accordance with the local legislation and institutional requirements. The participants provided their written informed consent to participate in this study.

## Author contributions

QS: Writing – review & editing, Writing – original draft, Visualization, Validation, Methodology, Formal analysis, Data curation, Conceptualization. AM: Writing – review & editing, Writing – original draft, Visualization, Validation, Methodology, Formal analysis, Data curation. MT: Writing – review & editing, Writing – original draft, Visualization, Validation, Supervision, Methodology, Funding acquisition, Formal analysis, Data curation, Conceptualization.
