# Supplementary material for: FOXP3 full length splice variant is associated with kidney allograft tolerance
Source: Front Immunol. 2024 Apr 10;15:1389105. doi: 10.3389/fimmu.2024.1389105 (PMC11040551; doi:10.3389/fimmu.2024.1389105)
Supplement: Supplementary file 1 [file DataSheet_1.docx]

**Title:**

FOXP3 Full Length Splice Variant is Associated with Kidney Allograft Tolerance

**Authors:**

Qais W. Saleh,^1,2^ Afsaneh Mohammadnejad^3^, Martin Tepel^1,2^

**Supplemental material table of contents**

Supplemental Table 1 – Details of primers used in quantitative polymerase chain reaction.

Supplemental Table 2 – Comparison of quantitative reverse transcription results when using 5 and 2 µl cDNA.

Supplemental Table 3 – Inter-sample variability.

Supplemental Table 4 – Intra-sample variability.

Supplemental Table 5 – Primer sets used for attempts to measure FOXP3 lacking exon 7.

Supplemental Table 6 – Danish ICD-10 diagnose codes used to determine patients that experienced rejection episodes.

Supplemental Table 7 – Missing data in FOXP3 splice variant measurements in included kidney transplant recipients.

Supplemental Table 8 – Potential confounding factors of the association of FOXP3 and declining eGFR in kidney transplant recipients.

Supplemental Table 9 – Peri-, and post-transplant characteristics for screened, included, and excluded patients.

Supplemental Table 10 – FOXP3 splice variant levels in screened, excluded -, and included patients.

Supplemental Table 11 – Model appropriateness test results according to tested exposure variable of interest.

Supplemental Table 12 – Odds ratio for co-variates in multivariable logistic regression analysis according to exposure variable of interest measured at day 1 post-transplant.

Supplemental Table 13 – Odds ratio of co-variates in multivariable logistic regression analysis according to exposure variable of interest measured at day 29 post-transplant.

Supplemental Figure 1 – Electrophoresis result of polymerase chain reaction product of pre-mRNA FOXP3, Total FOXP3, FOXP3fl, FOXP3d2, and β-actin.

Supplemental Figure 2 – Standard curves, and examples of amplification curves and melting peak of pre-mRNA FOXP3, total FOXP3, FOXP3fl, FOXP3d2, and β-actin.

Supplemental Figure 3 – Results of primer pairs used to measure FOXP3 lacking exon 2 and exon 7.

Supplemental Figure 4 – Distribution of logarithmic values of FOXP3 splice variants normalized to β-actin according to day of sample collection.

Supplemental Figure 5 – Association of day 29 pre-mRNA FOXP3 with a decline in eGFR within the first post-transplant year in kidney transplant recipients.

Supplemental Figure 6 – Ad hoc analysis results comparing pre-transplant FOXP3 splice variant levels with post-transplant levels.

**Supplemental Table 1 – Details of primers used in quantitative polymerase chain reaction.**

| Gene | Type | Sequence (5’-3’) | Product length (basepairs) | Annealing temperature (^o^ C) | Efficiency  (Using 5 ul cDNA) | Efficiency  (Using 2 ul cDNA) |
| --- | --- | --- | --- | --- | --- | --- |
| Pre-mRNA FOXP3 | Forward | TTCACCTGTGTATCTCACGCA | 352 | 63 | 1.97 |  |
|  | Reverse | gacagcggaggaagtagcta |  |  |  |  |
| Total FOXP3 | Forward | GTGGCCCGGATGTGAGAAG | 238 | 63 | 2.02 |  |
|  | Reverse | GGAGCCCTTGTCGGATGATG |  |  |  |  |
| β-actin | Forward | GGACTTCGAGCAAGAGATGG | 234 | 63 | 2.01 | 1.99 |
|  | Reverse | AGCACTGTGTTGGCGTACAG |  |  |  |  |
| FOXP3fl | Forward | AAAGCCTCAGACCTGCTG | 154 | 63 |  | 1.85 |
|  | Reverse | AGGGTGCCACCATGACTA |  |  |  |  |
| FOXP3d2 | Forward | CAGCTGCAGCTCTCAACGGTG | 128 | 63 |  | 1.95 |
|  | Reverse | GCCTTGAGGGAGAAGACC |  |  |  |  |

**Supplemental Table 2 – Comparison of quantitative reverse transcription results when using 5 and 2 µl cDNA.**

| Variant | Median logarithmic value normalized to β-actin when 5 µl cDNA is used | Median logarithmic value normalized to β-actin when 2 µl cDNA is used | Wilcoxon signed rank test |
| --- | --- | --- | --- |
| Total FOXP3 | -3.63 [-3.69 to -3.53] | -3.56 [-3.62 to -3.47] | 0.43 |
| Pre-mRNA FOXP3 | -4.48 [-4.55 to -4.48] | -4.37 [-4.58 to -4.35] | 0.22 |
| FOXP3fl | -4.45 [-4.54 to -4.37] | -4.49 [-4.63 to -4.43] | 0.68 |

We performed quantitative reverse transcription in 5 samples using 5 and 2 µl cDNA, the results were normalized to β-actin and compared with Wilcoxon signed rank test.

**Supplemental Table 3 – Inter-sample variability.**

| Variant | Cq values | | | Normalized logarithmic values | |
| --- | --- | --- | --- | --- | --- |
|  | Mean | Standard deviation | Coefficient of variation (CV) (%) | Mean | Standard deviation |
| Total FOXP3 | 29.44 | 0.44 | 1.49 | -4.30 | 0.15 |
| Pre-mRNA | 32.79 | 0.37 | 1.14 | -4.80 | 0.12 |
| FOXP3fl | 37.07 | 0.39 | 1.06 | -4.84 | 0.10 |
| FOXP3d2 | 32.16 | 0.28 | 0.89 | -4.26 | 0.07 |
| β-actin | 16.7 | 0.42 | 0.42 |  |  |

**Supplemental Table 4 – Intra-sample variability.**

| Variant | Cq values | | | Logarithmic values normalized to Β-actin | |
| --- | --- | --- | --- | --- | --- |
|  | Mean | Standard deviation | Coefficient of variation (CV) (%) | Mean | Standard deviation |
| Total FOXP3 | 29.48 | 0.89 | 3.04 | -4.38 | 0.13 |
| Pre-mRNA | 33.03 | 0.47 | 1.43 | -4.95 | 0.11 |
| FOXP3fl | 35.09 | 2.12 | 6.04 | -4.38 | 0.46 |
| FOXP3d2 | 31.23 | 1.49 | 4.78 | -4.06 | 0.30 |
| β-actin | 16.46 | 0.49 | 3.02 |  |  |

**Supplemental Table 5 – Primer sets used for attempts to measure FOXP3 lacking exon 7.**

| Name | Forward | Reverse | Product bp |
| --- | --- | --- | --- |
| 1 | 5 ’ GAGCAGCAGGCATCATCCG | 5’ CTGGGAATGTGCTGTTTCC | 160 |
| 2 |  | 5’ ATTGAGTGTCCGCTGCTT | 276 |
| 3 |  | 5’ GAAGGCAAACATGCGTGT | 312 |
| 4 | 5’ TGGAGCAGCAGGCATCAT | 5’ CTGGGAATGTGCTGTTTCC | 162 |
| 5 |  | 5’ ATTGAGTGTCCGCTGCTT | 278 |
| 6 |  | 5’ GAAGGCAAACATGCGTGT | 314 |
| 7 | 5’ GAGCAGCAGGCATCATC | 5’ CTGGGAATGTGCTGTTTCC | 160 |
| 8 |  | 5’ ATTGAGTGTCCGCTGCTT | 276 |
| 9 |  | 5’ GAAGGCAAACATGCGTGT | 312 |

All primer pairs produced a single product in samples from healthy people, but tended to produce more than one product in kidney transplant recipients, probably because of the low abundance of this variant in these patients.

**Supplemental Table 6 – Danish ICD-10 diagnose codes used to determine patients that experienced rejection episodes.**

| Diagnose Code | Translation |
| --- | --- |
| DT861 | Kidney transplant failure or rejection |
| DT861B | Kidney transplant rejection |
| DT868 | Failure or rejection of transplanted organ or tissue |
| DT869 | Failure or rejection of transplanted organ or tissue without further specification |

**Supplemental Table 7 – Missing data in FOXP3 splice variant measurements in included kidney transplant recipients.**

| Day of sample collection | FOXP3 splice variant | All  (N= 333) | Stable eGFR  (N = 201) | Declining eGFR  (N = 132) | p-value |
| --- | --- | --- | --- | --- | --- |
| One day post-transplant | Total FOXP3 | 26 (7.8 %) | 16 (8 %) | 10 (7.6 %) | 0.90 |
|  | Pre-mRNA FOXP3 | 39 (12 %) | 23 (11 %) | 16 (12 %) | 0.90 |
|  | FOXP3fl | 39 (12 %) | 24 (12%) | 15 (11 %) | 0.90 |
|  | FOXP3d2 | 36 (11 %) | 21 (10 %) | 15 (11 %) | 0.80 |
| 29 days post-transplant | Total FOXP3 | 42 (13 %) | 22 (11 %) | 20 (15 %) | 0.30 |
|  | Pre-mRNA FOXP3 | 62 (19 %) | 35 (17 %) | 27 (20 %) | 0.50 |
|  | FOXP3fl | 67 (20 %) | 37 (18 %) | 30 (23 %) | 0.30 |
|  | FOXP3d2 | 64 (19 %) | 36 (18 %) | 28 (21 %) | 0.50 |

FOXP3 splice variant levels are logarithmic values of FOXP3 splice variants normalized to β-actin. Data is presented as number of missing values (percent). There was no missing data in the variables: recipient age, - sex, donor type, use of IL-2 receptor antagonists, use of thymoglobulin, use of corticosteroid induction, first – vs. re-transplantation and cause of kidney disease. Stable eGFR: recipients with a difference in baseline eGFR (highest value of eGFR within 76-104 days post-transplant) and follow-up eGFR (highest eGFR within 337-365 days post-transplant < 5 ml/min/1.73m^2^. Declining eGFR: recipients with a difference in baseline eGFR and follow-up eGFR > 5 ml/min/1.73m^2^. P-value indicates result of Pearsons chi squared test comparing number of missing values in stable - and declining eGFR groups.

**Supplemental Table 8 – Potential confounding factors of the association of FOXP3 and declining allograft function in kidney transplant recipients.**

| Potential confounding variable | Articles reporting association to FOXP3 | Articles reporting association to declining allograft function or graft survival |
| --- | --- | --- |
| Recipient age | (1-3) | (4-6) |
| Recipient sex | (7) | (4, 5) |
| Donor type | (8) | (9) |
| Re-transplantation | (8) | (10, 11) |
| Cause of kidney disease | (12) | (13, 14) |
| Use of IL2-receptor antibodies (Basiliximab) | (15-17) | (18-20) |
| Use of thymoglobulin | (16, 21, 22) | (20, 23, 24) |
| Use of corticosteroids induction therapy | (25, 26) | (20, 27) |

These factors have reported association to FOXP3 and to declining allograft function by respective articles/guidelines/reviews. FOXP3: Forkhead box P3 transcription factor.

**References**

1. Garg SK, Delaney C, Toubai T, Ghosh A, Reddy P, Banerjee R, et al. Aging is associated with increased regulatory T-cell function. Aging Cell. 2014;13(3):441-8.

2. Palatella M, Guillaume SM, Linterman MA, Huehn J. The dark side of Tregs during aging. Front Immunol. 2022;13:940705.

3. Thomas AL, Alarcon PC, Divanovic S, Chougnet CA, Hildeman DA, Moreno-Fernandez ME. Implications of Inflammatory States on Dysfunctional Immune Responses in Aging and Obesity. Front Aging. 2021;2:732414.

4. Vinson AJ, Zhang X, Dahhou M, Süsal C, Döhler B, Sapir-Pichhadze R, et al. Age-dependent Sex Differences in Graft Loss After Kidney Transplantation. Transplantation. 2022;106(7):1473-84.

5. Hamilton AJ, Plumb LA, Casula A, Sinha MD. Associations with kidney transplant survival and eGFR decline in children and young adults in the United Kingdom: a retrospective cohort study. BMC Nephrology. 2020;21(1):492.

6. Hiramitsu T, Tomosugi T, Futamura K, Okada M, Matsuoka Y, Goto N, et al. Adult Living-Donor Kidney Transplantation, Donor Age, and Donor–Recipient Age. Kidney International Reports. 2021;6(12):3026-34.

7. Singh RP, Bischoff DS. Sex Hormones and Gender Influence the Expression of Markers of Regulatory T Cells in SLE Patients. Front Immunol. 2021;12:619268.

8. Saleh QW, Nagaraj S, Rasmussen M, Tepel M. Thymoglobulin reduces regulatory T cell specific forkhead box P3 (FOXP3) transcripts in kidney transplant recipients. 20222022.

9. Poggio ED, Augustine JJ, Arrigain S, Brennan DC, Schold JD. Long-term kidney transplant graft survival-Making progress when most needed. Am J Transplant. 2021;21(8):2824-32.

10. Magee JC, Barr ML, Basadonna GP, Johnson MR, Mahadevan S, McBride MA, et al. Repeat organ transplantation in the United States, 1996-2005. Am J Transplant. 2007;7(5 Pt 2):1424-33.

11. Trébern-Launay K, Foucher Y, Giral M, Legendre C, Kreis H, Kessler M, et al. Poor long-term outcome in second kidney transplantation: a delayed event. PLoS One. 2012;7(10):e47915.

12. Hu M, Wang YM, Wang Y, Zhang GY, Zheng G, Yi S, et al. Regulatory T cells in kidney disease and transplantation. Kidney Int. 2016;90(3):502-14.

13. Uffing A, Hullekes F, Riella LV, Hogan JJ. Recurrent Glomerular Disease after Kidney Transplantation: Diagnostic and Management Dilemmas. Clinical Journal of the American Society of Nephrology. 2021;16(11):1730-42.

14. Aleid H, Hammad E, Ibrahim I, Ali T, Raza S, AlManea H. Recurrent Glomerulonephritis after Kidney Transplantation in Saudi Arabia, Single Center Retrospective Study. Transplantation. 2018;102:S631.

15. Bouvy AP, Klepper M, Kho MM, Boer K, Betjes MG, Weimar W, et al. The impact of induction therapy on the homeostasis and function of regulatory T cells in kidney transplant patients. Nephrol Dial Transplant. 2014;29(8):1587-97.

16. Krystufkova E, Sekerkova A, Striz I, Brabcova I, Girmanova E, Viklicky O. Regulatory T cells in kidney transplant recipients: the effect of induction immunosuppression therapy. Nephrology Dialysis Transplantation. 2011;27(6):2576-82.

17. López-Abente J, Martínez-Bonet M, Bernaldo-de-Quirós E, Camino M, Gil N, Panadero E, et al. Basiliximab impairs regulatory T cell (TREG) function and could affect the short-term graft acceptance in children with heart transplantation. Sci Rep. 2021;11(1):827.

18. Chapman TM, Keating GM. Basiliximab: a review of its use as induction therapy in renal transplantation. Drugs. 2003;63(24):2803-35.

19. McKeage K, McCormack PL. Basiliximab: a review of its use as induction therapy in renal transplantation. BioDrugs. 2010;24(1):55-76.

20. KDIGO clinical practice guideline for the care of kidney transplant recipients. Am J Transplant. 2009;9 Suppl 3:S1-155.

21. Tang Q, Leung J, Melli K, Lay K, Chuu EL, Liu W, et al. Altered balance between effector T cells and FOXP3+ HELIOS+ regulatory T cells after thymoglobulin induction in kidney transplant recipients. Transpl Int. 2012;25(12):1257-67.

22. Krepsova E, Tycova I, Sekerkova A, Wohlfahrt P, Hruba P, Striz I, et al. Effect of induction therapy on the expression of molecular markers associated with rejection and tolerance. BMC Nephrol. 2015;16:146.

23. Hill P, Cross NB, Barnett AN, Palmer SC, Webster AC. Polyclonal and monoclonal antibodies for induction therapy in kidney transplant recipients. Cochrane Database Syst Rev. 2017;1(1):Cd004759.

24. Lasmar MF, Dutra RS, Nogueira-Machado JA, Fabreti-Oliveira RA, Siqueira RG, Nascimento E. Effects of immunotherapy induction on outcome and graft survival of kidney-transplanted patients with different immunological risk of rejection. BMC Nephrology. 2019;20(1):314.

25. Kim D, Nguyen QT, Lee J, Lee SH, Janocha A, Kim S, et al. Anti-inflammatory Roles of Glucocorticoids Are Mediated by Foxp3(+) Regulatory T Cells via a miR-342-Dependent Mechanism. Immunity. 2020;53(3):581-96.e5.

26. Karagiannidis C, Akdis M, Holopainen P, Woolley NJ, Hense G, Rückert B, et al. Glucocorticoids upregulate FOXP3 expression and regulatory T cells in asthma. J Allergy Clin Immunol. 2004;114(6):1425-33.

27. Steiner RW, Awdishu L. Steroids in kidney transplant patients. Semin Immunopathol. 2011;33(2):157-67.

**Supplemental Table 9 – Peri-, and post-transplant characteristics for screened, included, and excluded patients.**

| Characteristic | All patients  (N = 617) | Included patients  (N = 333) | Excluded  patients  (N = 284) | P-value |
| --- | --- | --- | --- | --- |
| *Recipient Data* | | | | |
| Age (years) | 52 [41 to 62] | 54 [43 to 63] | 51 [40 to 61] | 0.06^a^ |
| Male sex, N (%) | 407 (66%) | 216 (65 %) | 191 (67 %) | 0.50^c^ |
| Height (cm) | 175 [168 to 182] | 174 [167 to 182] | 176 [168 to 183] | 0.20^a^ |
| Weight (kg) | 81 [70 to 93] | 81 [70 to 93] | 82 [70 to 92] | 0.60^a^ |
| Diabetes, N (%) | 111 (18%) | 67 (20 %) | 44 (15 %) | 0.14^c^ |
| Coronary artery disease, N (%) | 57 (9.2%) | 33 (9.9 %) | 24 (8.5 %) | 0.50^c^ |
| Active tobacco use, N (%) | 173 (28%) | 96 (29 %) | 77 (27 %) | 0.60^c^ |
| Cause of kidney disease, N (%)  Glomerulonephritis  Diabetic nephropathy  Hypertensive nephropathy  Other  Unknown | 212 (34 %)  90 (15 %)  84 (14 %)  132 (21 %)  99 (16 %) | 106 (31 %)  54 (16 %)  42 (13 %)  72 (22 %)  59 (18 %) | 106 (37 %)  36 (13 %)  42 (15 %)  60 (21 %)  40 (14 %) | 0.30^b^ |
| Duration of dialysis (months) | 12 (2, 30) | 12 (2, 30) | 12 (3, 30) | 0.50^a^ |
| Type of dialysis, N (%)  Pre-emptive  Hemodialysis  Peritoneal dialysis | 129 (21 %)  331 (24 %)  157 (25 %) | 75 (23 %)  181 (54 %)  77 (23%) | 54 (19 %)  150 (53 %)  80 (28 %) | 0.30^b^ |
| Transplantation, N (%)  First transplant  Second/more | 534 (87 %)  83 (13 %) | 283 (85 %)  50 (15 %) | 251 (88 %)  33 (12 %) | 0.20^c^ |
| Donor type, N (%)  Deceased  Living ABO-compatible  Living ABO-incompatible | 348 (56 %)  192 (31%)  77 (13%) | 191 (57 %)  99 (30 %)  43 (13 %) | 157 (55 %)  93 (33 %)  34 (12 %) | 0.70^b^ |
| Number of HLA mismatches (range within 0-8) | 3 [2 to 4] | 3 [2 to 4] | 3 [2 to 4] | 0.07^a^ |
| Delayed graft function, N (%) | 78 (13 %) | 47 (14 %) | 31 (11 %) | 0.20^c^ |
| Plasma creatinine pre-transplant (µmol/l) | 710 [552 to 890] | 710 [552 to 890] | 734 [556 to 912] | 0.09^a^ |
| Experienced at least 1 rejection episode within the first post-transplant year | 119 (19 %) | 70 (21 %) | 49 (17 %) | 0.20^c^ |
| *Induction and Maintenance Therapy* | | | | |
| IL2-receptor antibodies (*Basiliximab*), N (%) | 501 (81%) | 272 (82 %) | 229 (81 %) | 0.70^c^ |
| Thymoglobulin, N (%) | 94 (15%) | 52 (16 %) | 42 (15 %) | 0.80^c^ |
| Corticosteroids, N (%) | 177 (29%) | 92 (28 %) | 85 (30 %) | 0.50^c^ |
| Anti-CD20 antibodies (*Rituximab)*, N (%) | 122 (20%) | 69 (21 %) | 53 (19 %) | 0.50^c^ |
| Maintenance therapy, N (%)  Tacrolimus  Cyclosporine  Mycofenolate | 615 (99.7 %)  2 (0.3 %)  617 (100%) | 333 (100 %)  0  333 (100 %) | 282 (99 %)  2 (0.7 %)  284 (100 %) | 0.20^b^ |
| *Living Donor Data* | | | | |
| Age | 54 [45 to 63]  (Missing = 171) | 55 [46 to 64]  (Missing = 85) | 52 [44 to 62]  (Missing = 86) | 0.03 ^a^ |
| Male sex, N (%) | 191 (42%)  (Missing = 164) | 107 (42 %)  (Missing = 81) | 84 (42 %)  (Missing = 83) | 0.90^c^ |
| Cold ischemic time (minutes) | 780 [589 to 1020]  (Missing = 285) | 780 [585 to 1080]  (Missing = 156) | 780 [600 to 1013]  (Missing = 129) | 0.30^a^ |

Other causes of end stage kidney disease encompass hydronephrosis, cancer and polycystic kidney disease. HLA: human leukocyte antigen. Numerical data is presented as median [interquartile range] and tested with Wilcoxon rank sum test (a), categorical data is presented as number (percent) and tested with Pearson’s Chi squared test (b) or Fischer’s exact test (c) as appropriate.

**Supplemental Table 10 – FOXP3 splice variant levels in screened, excluded -, and included patients.**

| Day of sample collection post-transplant | Variant | Screened  (N = 617) | Excluded  (N = 284) | Included  (N = 333) | P-value |
| --- | --- | --- | --- | --- | --- |
| One | Total FOXP3 | -3.70 [-4.00 to ‑3.40] | -3.72 [-4.00 to ‑3.45] | -3.67 [-3.98 to ‑3.36] | 0.20 |
|  | Pre-mRNA FOXP3 | -4.42 [-4.69 to ‑4.11] | -4.40 [-4.65 to ‑4.06] | -4.46 [-4.74 to ‑4.16] | 0.06 |
|  | FOXP3fl | -4.01 [-4.34 to ‑3.76] | -4.04 [-4.37 to ‑3.77] | -3.94 [-4.32 to ‑3.74] | 0.40 |
|  | FOXP3d2 | -3.76 [-4.03 to ‑3.52] | -3.78 [-4.04 to ‑3.53] | -3.72 [-4.00 to ‑3.52] | 0.40 |
| 29 | Total FOXP3 | -3.60 [-3.90 to ‑3.33] | -3.62 [-3.91 to ‑3.36] | -3.56 [-3.86 to ‑3.28] | 0.08 |
|  | Pre-mRNA FOXP3 | -4.35 [-4.63 to ‑4.06] | -4.37 [-4.64 to ‑4.11] | -4.34 [-4.62 to ‑4.04] | 0.40 |
|  | FOXP3fl | -3.87 [-4.19 to ‑3.63] | -3.89 [-4.22 to ‑3.64] | -3.80 [-4.15 to ‑3.61] | 0.11 |
|  | FOXP3d2 | 3.69 [-3.97 to ‑3.44] | -3.70 [-3.97 to ‑3.45] | -3.64 [-3.96 to ‑3.43] | 0.30 |

Data are logarithmic values of FOXP3 splice variants normalized to β-actin. Numerical data is presented as median [interquartile range] and tested with Wilcoxon rank sum test.

**Supplemental Table 11 – Model appropriateness test results according to tested exposure variable of interest.**

| Day of sample collection post-transplant | Exposure variable of interest | Stukels test p-value | Likelihood ratio test p-value |
| --- | --- | --- | --- |
| One | Total FOXP3 | 0.66 | 0.28 |
|  | Pre-mRNA FOXP3 | 0.21 | 0.21 |
|  | FOXP3fl | 0.69 | 0.02 |
|  | FOXP3d2 | 0.86 | 0.15 |
| 29 | Total FOXP3 | 0.99 | 0.51 |
|  | Pre-mRNA FOXP3 | 0.85 | 0.02 |
|  | FOXP3fl | 0.99 | 0.70 |
|  | FOXP3d2 | 0.80 | 0.70 |

**Supplemental Table 12 – Odds ratio for co-variates in multivariable logistic regression analysis according to exposure variable of interest measured at day 1 post-transplant.**

|  | *Exposure Variable of Interest* | | | |
| --- | --- | --- | --- | --- |
|  | Total FOXP3 | Pre-mRNA FOXP3 | FOXP3fl | FOXP3d2 |
| Covariate | OR (CI), p-value | OR (CI), p-value | OR (CI), p-value | OR (CI), p-value |
| Recipient age | 0.98 (0.98-1.01), 0.15 | 0.98 (0.96-1.01), 0.23 | 0.98 (0.96-1.01), 0.14 | 0.98 (0.96-1.01), 0.17 |
| Recipient male sex | 1.75 (1.03-2.97), 0.03 | 1.92 (1.10-3.33), 0.02 | 1.84 (1.06-3.19), 0.02 | 1.84 (1.07-3.18), 0.02 |
| Donor type  Deceased  Living ABO-compatible  Living ABO-incompatible | -  0.57 (0.31-1.03), 0.06  1.36 (0.43-4.25), 0.59 | -  0.58 (0.32-1.08), 0.08  1.61 (0.49-5.29), 0.42 | -  0.57 (0.31-1.06), 0.07  1.44 (0.43-4.48), 0.55 | -  0.56 (0.30-1.02), 0.06  1.52 (0.45-5.07), 0.49 |
| Prior transplantation | 0.98 (0.46-2.07), 0.96 | 0.95 (0.44-2.07), 0.91 | 0.88 (0.41-1.88), 0.74 | 0.93 (0.44-1.98), 0.86 |
| Cause of kidney disease  Diabetic nephropathy  Glomerulonephritis  Hypertensive nephropathy  Other  Unknown | -  0.74 (0.35-1.58), 0.45  1.42 (0.61-3.34), 0.45  0.50 (0.22-1.12), 0.09  0.73 (0.39-1.65),  0.46 | -  0.73 (0.33-1.57), 0.42  1.44 (0.61-3.40), 0.40  0.49 (0.21-1.14), 0.09  0.71 (0.31-1.64), 0.43 | -  0.77 (0.35-1.68), 0.52  1.53 (0.64-3.68), 0.33  0.46 (0.20-1.06), 0.07  0.76 (0.33-1.75), 0.53 | -  0.80 (0.37-1.72), 0.57  1.55 (0.65-3.69), 0.31  0.50 (0.22-1.14), 0.10  0.80 (0.35-1.84), 0.61 |
| IL2-receptor antibodies (Basiliximab) | 0.50 (0.11-2.16), 0.35 | 0.48 (0.09-2.56), 0.39 | 0.59 (0.11-3.10), 0.53 | 0.60 (0.11-3.16), 0.55 |
| Corticosteroids | 0.48 (0.16-1.49), 0.20 | 0.40 (0.12-1.9), 0.12 | 0.50 (0.15-1.63), 0.25 | 0.49 (0.15-1.59), 0.23 |
| Thymoglobulin | 1.14 (0.21-6.00), 0.20 | 1.82 (0.28-11.67), 0.52 | 1.02 (0.14-7.05), 0.97 | 1.20 (0.17-8.26), 0.84 |

The independent variable is a decline in estimated glomerular filtration rate above 5 ml/min/1.73m^2^ from baseline (76 to 104 days after transplantation) until follow-up (337-365 days after transplantation). OR = odds ratio. CI = 95 % confidence interval. The subcategory “other” under cause of kidney disease include cystic kidney disease, hydronephrosis and cancer.

**Supplemental Table 13 – Odds ratio of co-variates in multivariable logistic regression analysis according to exposure variable of interest measured at day 29 post-transplant.**

|  | *Exposure Variable of Interest* | | | |
| --- | --- | --- | --- | --- |
|  | Total FOXP3 | Pre-mRNA FOXP3 | FOXP3fl | FOXP3d2 |
| Covariate | OR (CI), p-value | OR (CI), p-value | OR (CI), p-value | OR (CI), p-value |
| Recipient age | 0.99 (0.97-1.01), 0.55 | 0.99 (0.97-1.01), 0.62 | 0.99 (0.97-1.01), 0.72 | 0.99 (0.97-1.01), 0.68 |
| Recipient male sex | 1.53 (0.90-2.62), 0.11 | 1.52 (0.86-2.66), 0.14 | 1.50 (0.85-2.64), 0.15 | 1.48 (0.85-2.59), 0.16 |
| Donor type  Deceased  Living ABO-compatible  Living ABO-incompatible | -  0.57 (0.31-1.04), 0.07  1.19 (0.38-3.73), 0.75 | -  0.55 (0.29-1.04), 0.07  1.75 (0.49-6.23), 0.38 | -  0.55 (0.29-1.05), 0.07  1.82 (0.51-6.45), 0.35 | -  0.61 (0.32-1.14), 0.12  1.83 (0.51-6.47), 0.34 |
| Prior transplantation | 1.41 (0.68-2.93), 0.35 | 1.47 (0.67-3.24), 0.33 | 1.49 (0.67-3.32), 0.32 | 1.58 (0.72-3.46), 0.24 |
| Cause of kidney disease  Diabetic nephropathy  Glomerulonephritis  Hypertensive nephropathy  Other  Unknown | -  0.81 (0.37-1.79), 0.61  1.49 (0.57-3.53), 0.43  0.55 (0.24-1.25), 0.15  0.60 (0.24-1.46), 0.26 | -  0.76 (0.33-1.73), 0.51  1.38 (0.54-3.51), 0.48  0.49 (0.21-1.18), 0.11  0.56 (0.22-1.43), 0.22 | -  0.92 (0.39-2.13), 0.85  1.51 (0.58-3.89), 0.38  0.55 (0.23-1.33), 0.18  0.58 (0.22-1.50), 0.26 | -  0.77 (0.34-1.76), 0.54  1.33 (0.51-3.41), 0.55  0.50 (0.21-1.18), 0.11  0.57 (0.22-1.46), 0.24 |
| IL2-receptor antibodies (Basiliximab) | 0.32 (0.07-1.46), 0.14 | 0.41 (0.07-2.29), 0.31 | 0.39 (0.07-2.16), 0.28 | 0.37 (0.06-2.05), 0.25 |
| Corticosteroids | 0.42 (0.13-1.36), 0.15 | 0.29 (0.07-1.07), 0.06 | 0.28 (0.07-1.06), 0.06 | 0.27 (0.07-0.99), 0.04 |
| Thymoglobulin | 1.15 (0.23-5.75), 0.86 | 1.96 (0.29-12.95), 0.48 | 1.69 (0.25-11.40), 0.58 | 1.53 (0.22-10.35), 0.66 |

The independent variable is a decline in estimated glomerular filtration rate above 5 ml/min/1.73m^2^ from baseline (76 to 104 days after transplantation) until follow-up (337-365 days after transplantation). OR = odds ratio. CI = 95 % confidence interval. The subcategory “other” under cause of kidney disease include cystic kidney disease, hydronephrosis and cancer.

Supplemental Figure 1 – electrophoresis result of polymerase chain reaction product of pre-mRNA FOXP3, Total FOXP3, FOXP3fl, FOXP3d2, and β-actin. Bp: basepairs.


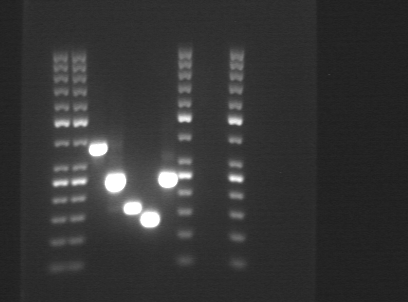


SD

SD

SD

SD

A

B

C

D

E

50 bp

250 bp

500 bp

SD – Standard

A – pre-mRNA FOXP3

B – Total FOXP3

C – FOXP3fl

D – FOXP3d2

E – β-actin

F – Reverse Transcriptase (-) control

G – H_2_O

F

G

Supplemental Figure 2 – Standard curves, and examples of amplification curves and melting peak of (A) pre-mRNA FOXP3, (B) total FOXP3, (C) FOXP3fl, (D) FOXP3d2, and (E) β-actin.

A - Pre-mRNA FOXP3


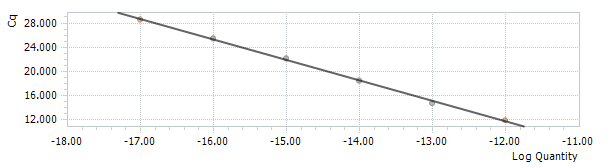

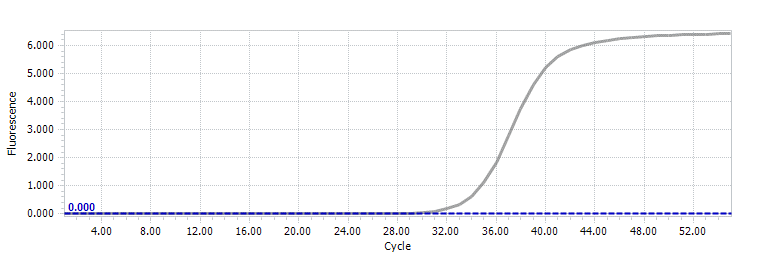

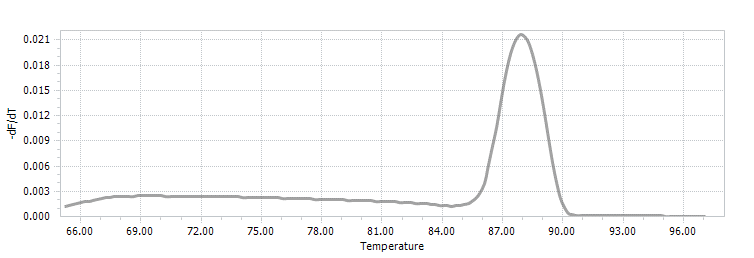


B - Total FOXP3


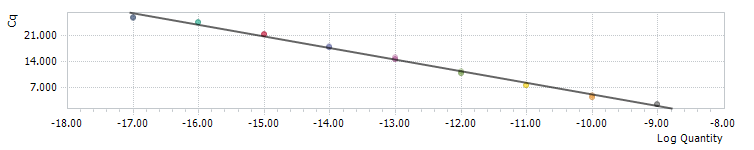

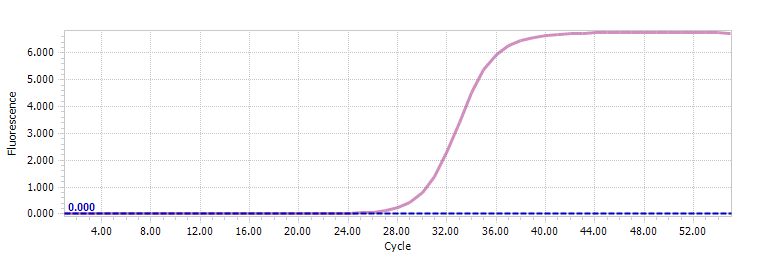

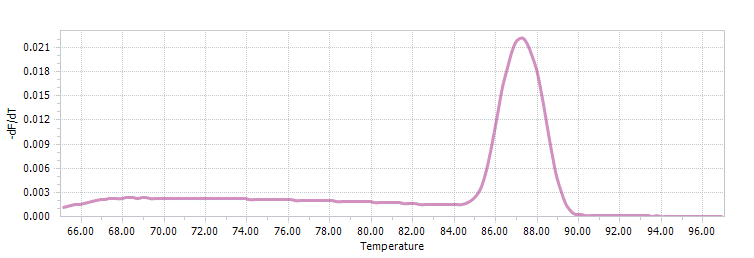


C - FOXP3fl


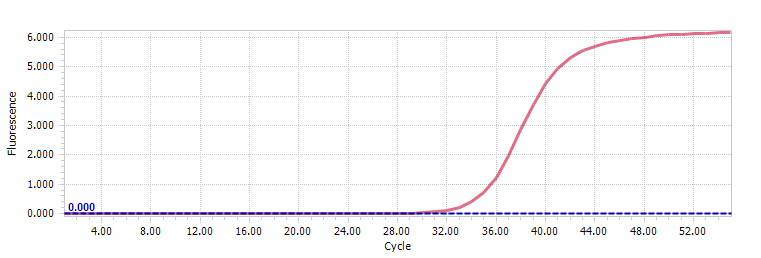

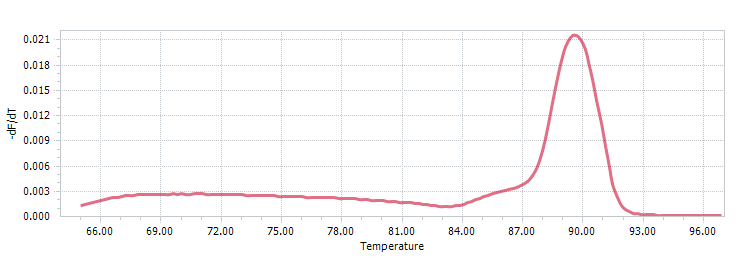

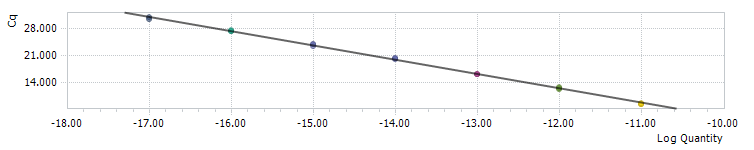

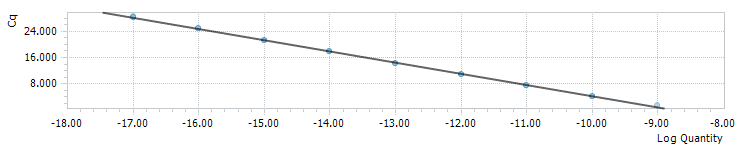

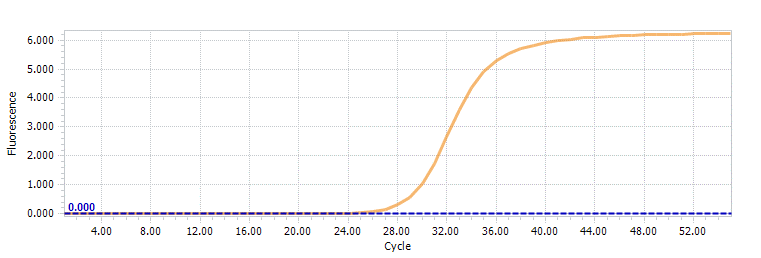

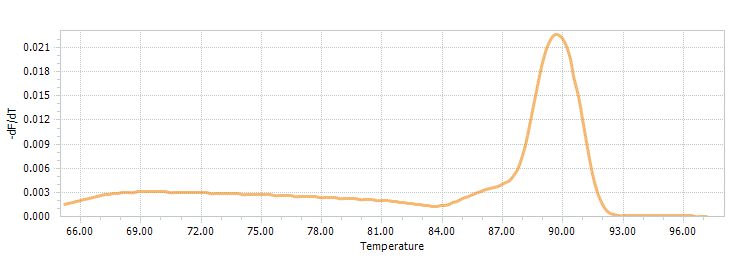


D - FOXP3d2

E – β-actin


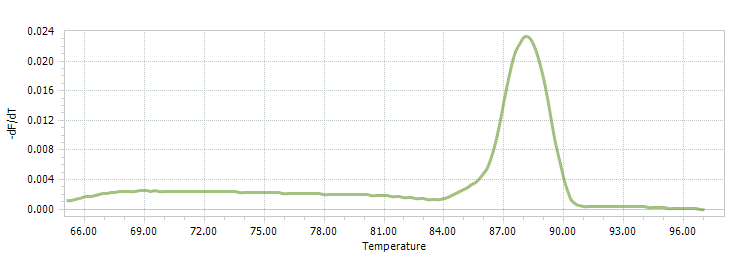

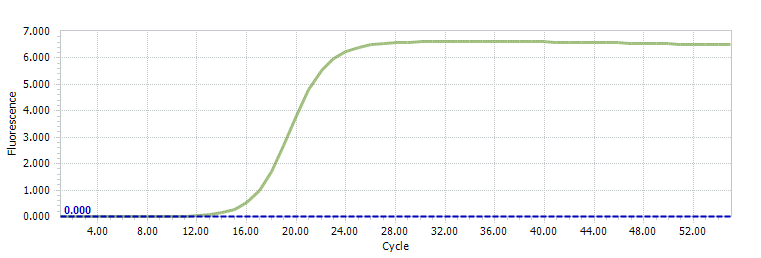

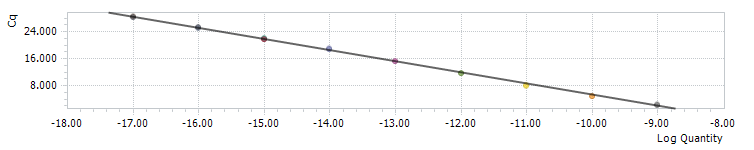


Supplemental Figure 3 – Results of primer pairs used to measure FOXP3 lacking exon 2 and exon 7, all primer details are displayed in Supplemental Table 5. A) we tested 9 primer pairs in samples from healthy donors, these showed that all primers produced one product in quantitative polymerase chain reaction. B) melting peaks of all tested primer pairs in samples of healthy donors, primer pairs 2 to 9 showed some irregularities. C) we tested primer pair 1 with multiple samples derived from kidney transplant recipients one day post-transplant, out of 9 samples tested, 5 (55 %) showed multiple bands. D) gel electrophoresis result for primer pairs 4, 5 and 6 in samples (a to e) derived from kidney transplant recipients one day post-transplant, these showed multiple bands. SD = standard ladder. Bp = basepairs.


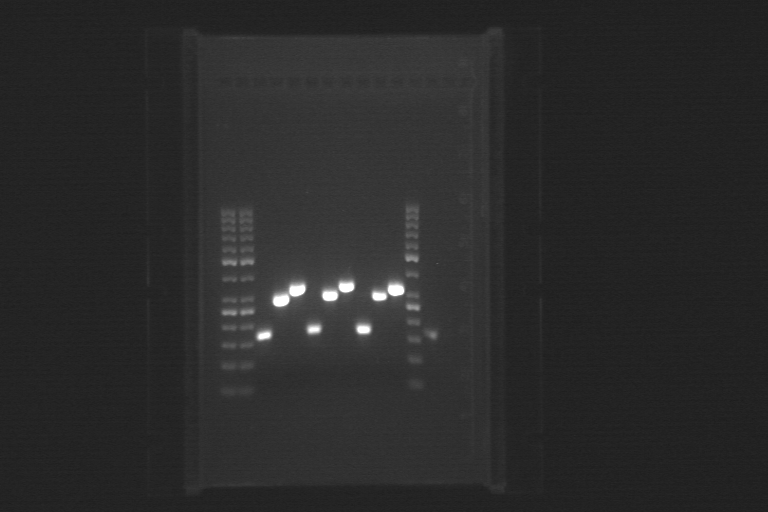


1

2

3

4

5

6

7

8

9

1

Healthy Donor Sample

Recipient sample

SD

SD

50 bp

250 bp

A


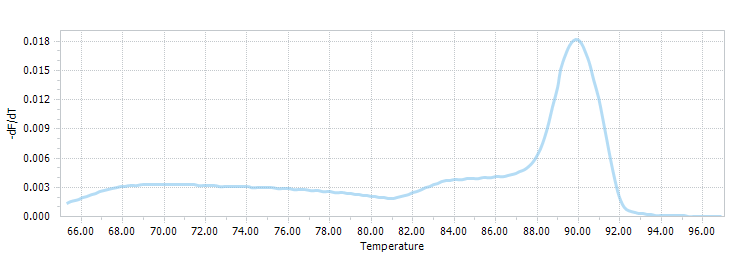

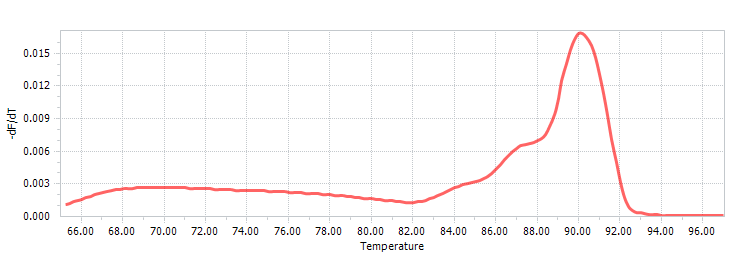

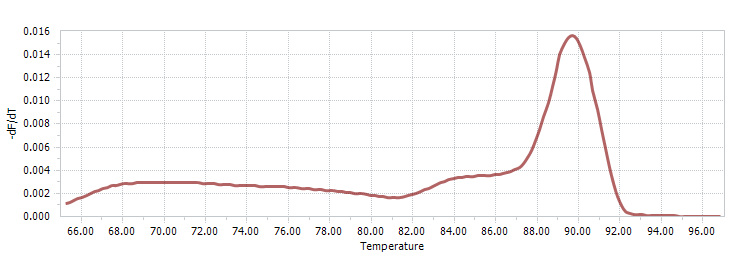

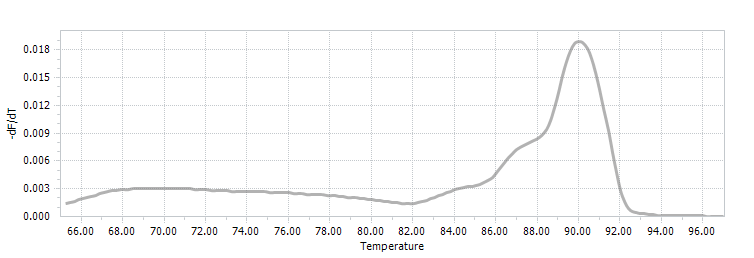

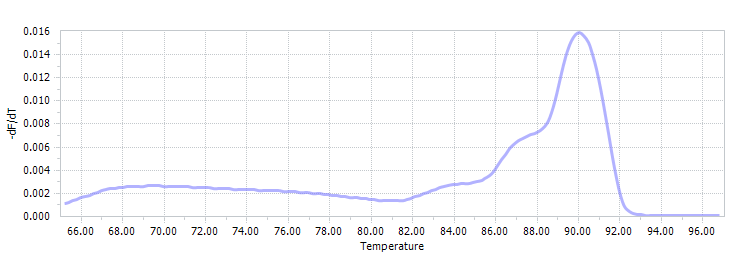

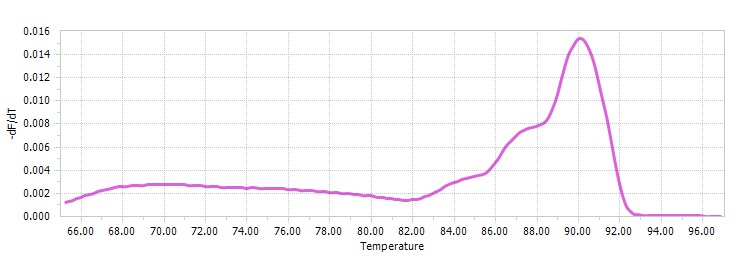

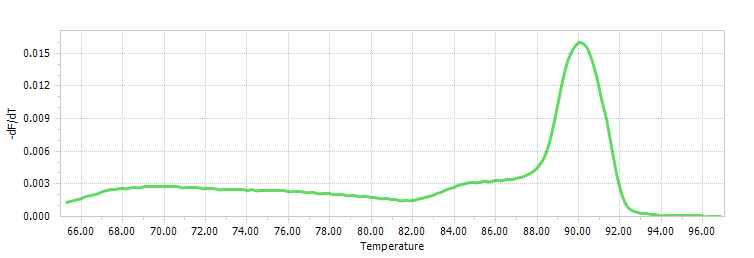

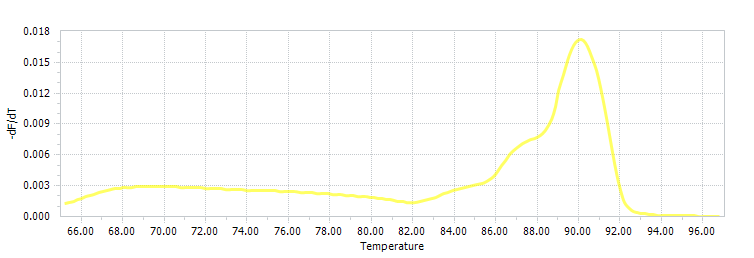

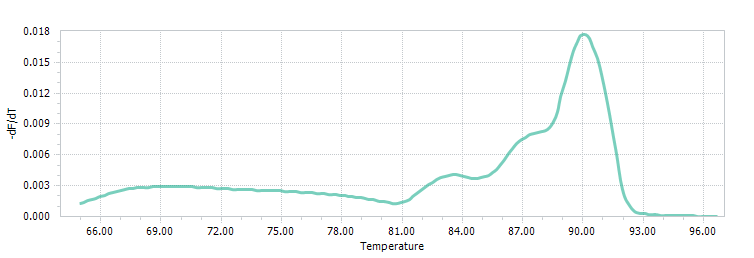


1

2

3

4

5

6

7

8

9

B


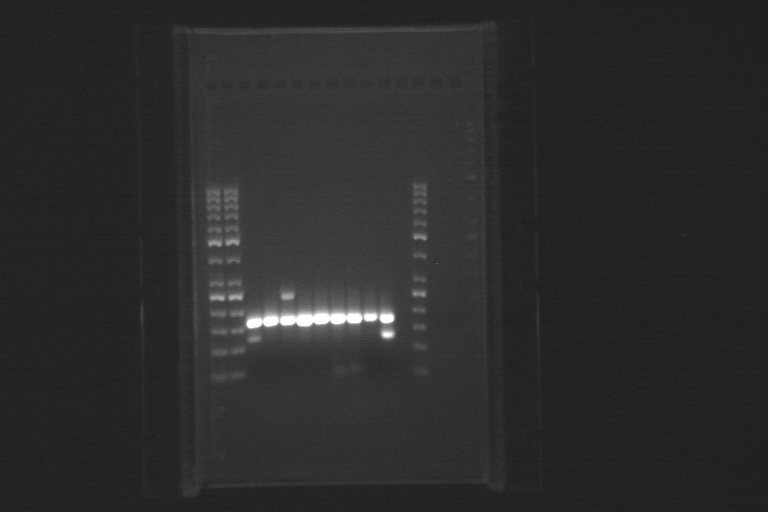


Primer pair 1

SD

SD

50 bp

250 bp

C


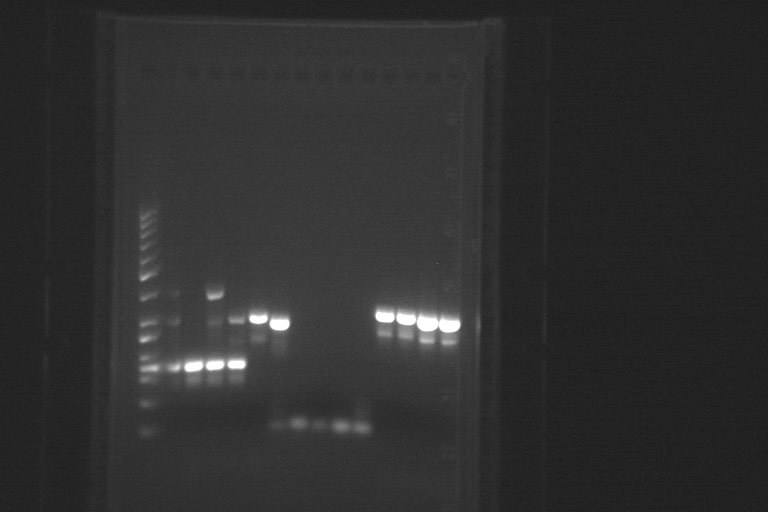


SD

50 bp

250 bp

Primer 4

Primer 5

Primer 6

a

b

c

d

e

a

b

c

d

e

a

b

c

d

D

8

Supplemental Figure 4 – Distribution of logarithmic values of FOXP3 splice variants normalized to β-actin according to day of sample collection. A) One day post-transplant, B) 29 days post-transplant. Vertical lines indicate median values. Total FOXP3: all mature splice variants, FOXP3fl: mature FOXP3 mRNA containing all exons, FOXP3d2: mature FOXP3 mRNA in which exon 2 is spliced. Declining eGFR: a decline from in estimated glomerular filtration rate from baseline (76 to 104 days post-transplant) to follow-up (337 to 365 days post-transplant) eGFR above 5 ml/min/1.73m2. Stable eGFR: the difference between baseline and follow-up eGFR is below 5 ml/min/1.73m2. There is a significant difference between median values of FOXP3fl in events measured at day 1 vs that of non-events measured at day 1, using Wilcoxon rank sum test, p = 0.02. Otherwise there were no statistically significant differences between groups.


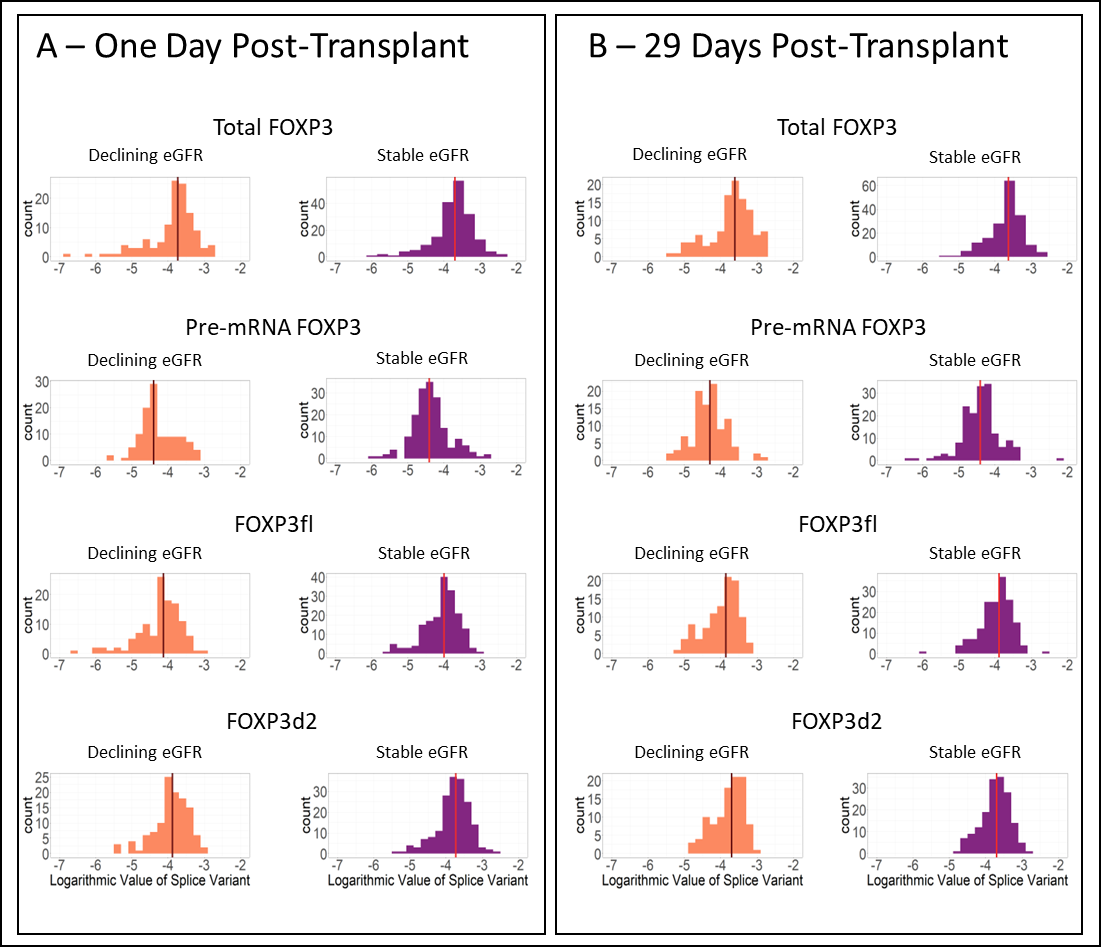


Supplemental Figure 5 – High day 29 pre-mRNA FOXP3 is independently associated with a decline in eGFR within the first post-transplant year in kidney transplant recipients. A) Distribution of logarithmic values of FOXP3fl normalized to β-actin, vertical lines indicate median values, there was not statistically significant difference between medians (Wilcoxon rank sum test, p = 0.11). B) Univariate logistic regression analysis results where FOXP3 spice variants measured 29 days post-transplant are the exposure variables of interest and a decline in eGFR within the first post-transplant year is the outcome. C) Multivariate Logistic regression analysis results where the exposure variable of interest is FOXP3 splice variants measured 29 days post-transplant, the outcome is a decline in eGFR within the first post-transplant year is the outcome, and the adjusted co-variates are: recipient age, recipient sex, donor type, prior transplantation, underlying cause of kidney disease and immunosuppressive induction therapy. D) Test of trend between pre-mRNA FOXP3 levels in quartiles and percent of included patients who experienced a decline in eGFR. eGFR: estimated glomerular filtration rate. Declining eGFR: a decrease from baseline (76 to 104 days post-transplant) to follow-up (337 to 365 days post-transplant) eGFR above 5 ml/min/1.73m^2^. Stable eGFR: the difference between baseline and follow-up eGFR is below 5 ml/min/1.73m^2^.


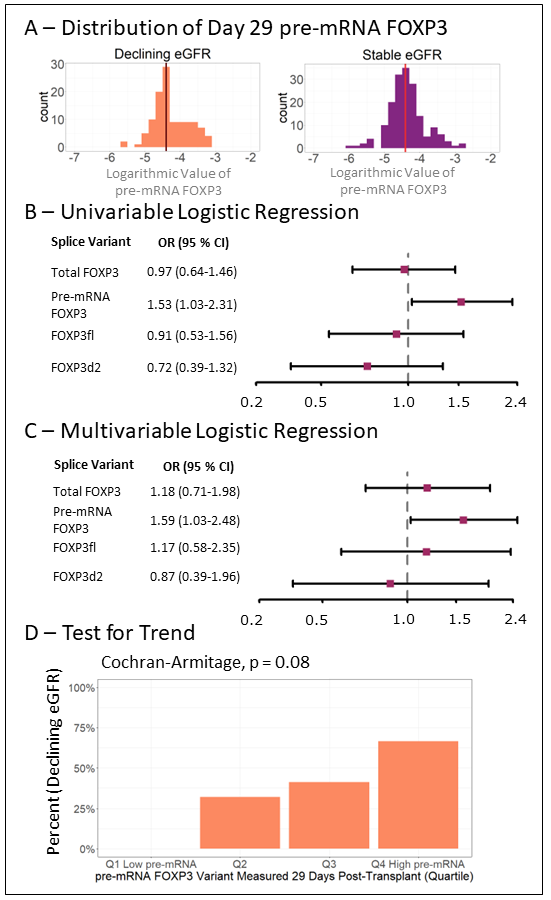


Supplemental Figure 6 – In ad hoc analysis we measured pre-transplant levels of FOXP3fl in included patients who had available pre-transplant samples (N = 150). These levels where compared in patients with stable- and declining one-year post-transplant eGFR. Data is shown as median and inter-quartile range. Stable eGFR: recipients with a difference in baseline eGFR (highest value of eGFR within 76-104 days post-transplant) and follow-up eGFR (highest eGFR within 337-365 days post-transplant < 5 ml/min/1.73m^2^. Declining eGFR: recipients with a difference in baseline eGFR and follow-up eGFR > 5 ml/min/1.73m^2^. Lines indicate result of comparison using signed rank test. *** indicate significant statistical difference in median (p < 0.01) of post-transplant FOXP3fl level compared to pre-transplant levels. Comparisons were done with two-tailed Wilcoxon signed rank test.
